# Ultrasound-assisted multicomponent synthesis of 4*H*-pyrans in water and DNA binding studies

**DOI:** 10.1038/s41598-020-68076-1

**Published:** 2020-07-14

**Authors:** Fernando Auria-Luna, Vanesa Fernández-Moreira, Eugenia Marqués-López, M. Concepción Gimeno, Raquel P. Herrera

**Affiliations:** 1 Departamento de Química Orgánica, Laboratorio de Organocatálisis Asimétrica, Instituto de Síntesis Química y Catálisis Homogénea (ISQCH), CSIC-Universidad de Zaragoza, C/ Pedro Cerbuna, Nº12, 50009 Zaragoza, Spain; 2 Departamento de Química Inorgánica, Instituto de Síntesis Química y Catálisis Homogénea (ISQCH), CSIC-Universidad de Zaragoza, C/ Pedro Cerbuna, Nº12, 50009 Zaragoza, Spain

**Keywords:** Biochemistry, Catalysis, Chemical biology, Chemical safety, Green chemistry, Organic chemistry, Chemical synthesis

## Abstract

A simple approach to synthesize new highly substituted 4*H*-pyran derivatives is described. Efficient Et_3_N acts as a readily accessible catalyst of this process performed in pure water and with only a 20 mol% of catalyst loading. The extremely simple operational methodology, short reaction times, clean procedure and excellent product yields render this new approach extremely appealing for the synthesis of 4*H*-pyrans, as potentially biological scaffolds. Additionally, DNA interaction analysis reveals that 4*H*-pyran derivatives behave preferably as minor groove binders over major groove or intercalators. Therefore, this is one of the scarce examples where pyrans have resulted to be interesting DNA binders with high binding constants (*K*_b_ ranges from 1.53 × 10^4^ M^−1^ to 2.05 × 10^6^ M^−1^).

## Introduction

Highly functionalized 4*H*-pyrans are an important family of oxygen-containing heterocycles with a wide spectrum of biological properties. The 4*H*-pyran core can be found in many natural products or pharmaceutical compounds, commonly as part of 4*H*-chromene skeletons (4*H*-1-benzopyrans)^1–3^. Their interesting pharmacological profile varies from antitumor, antiallergic, antimicrobial to antibacterial agent, among other properties^4–10^. By similarity with 1,4-dihydropyridines, 4*H*-pyrans have been also applied as calcium channel blockers^11^. Moreover, the use of this family of organic compounds has been extended to the cosmetic and agrochemical industry^12^.

**Figure 1:**
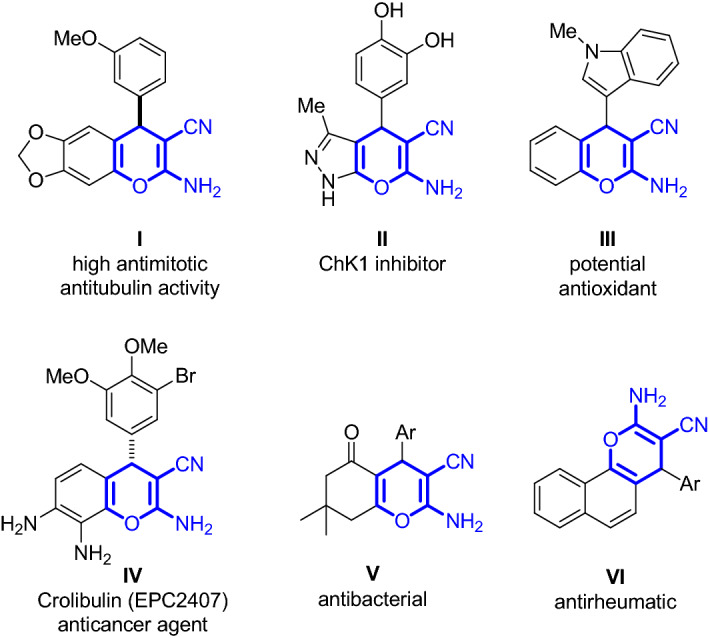
Biologically active 2-amino-3-cyanopyran structural cores as part of 2-amino-3-cyano-4*H*-chromene skeletons: **I**^13^, **II**^14^, **III**^15^, **IV**^16^, **V**^17^ and **VI**^18^.

Among them, the synthesis of 2-amino-3-cyanopyran derivatives has aroused special attention in the last few years, as part of 2-amino-3-cyano-4*H*-chromenes^3^, also because of their biological properties (Fig. 1).

Because of the importance of these compounds, their synthesis has been an active task in organic chemistry for a long time^19^. 4*H*-Pyrans have been synthesized following diverse methodologies, although in most of these examples the core is contained in a more complex chromene structure. Recent preparations of 4*H*-pyrans involved more complex catalysts such as ionic liquids^20^, heterogeneous catalysts^21^, polyethylene glycol (PEG)^22^, magnetic nanoparticles^23–26^, MOFs^27^ or sophisticated organocatalysts^28^, among many others. Therefore, the development of new simple, efficient and economical procedures affording 4*H*-pyrans is still required.

In addition, with the growing concern about sustainability, the use of water as a solvent or co-solvent has become one of the main challenges in green chemistry, for being the most environmentally friendly medium^29^. However, because of the general poor solubility of organic compounds in water, or the high reactivity of some reagents in this medium, the use of water to perform organic syntheses had been eluded for a long time. In contrast, nowadays the interest in water as reaction medium has been increased due to its attractive practical advantages over other solvents for its accessibility and safety and from an economic and environmental point of view^30–39^. Furthermore, the advantages of using water would be in agreement with some of the twelve principles of Green Chemistry^40–44^. However, despite the number of processes that have been investigated and developed in water, maybe because of the good solubility of reactants is generally considered a prerequisite for an appropriate reactivity, water is still not commonly used as a sole solvent for organic reactions^45–49^. For all these reasons, the development of reactions in pure water is still a challenging task.

Multicomponent methodologies have attracted the efforts of many research groups^50–52^ due to their potential for efficient construction of highly complex molecules in a single reaction step, avoiding difficult purification operations and allowing savings of both solvents and reagents. Therefore, the development of new multicomponent protocols in water affording more complex structures with higher synthetic values is of great interest and an active challenge in organic synthesis^53–56^.

In the same context, with the aim of developing new synthetic reactions or accelerate them, overcoming the activation energy, unconventional energy sources have been employed^57–64^. In particular, ultrasounds have been used in many transformations. Although this source of energy is a field in continuous growth in organic chemistry, in general, its use in multicomponent reactions has been less explored so far^65–67^.

In agreement with all these aspects, we report the development of more efficient and sustainable protocols for the synthesis of highly functionalized 4*H*-pyrans via base catalysis. Moreover, and because of the interesting biological activity shown by this type of scaffolds, the study of their DNA interactions is also reported.

## Results and discussion

### Synthesis of 4*H*-pyrans in water

**Figure 2:**
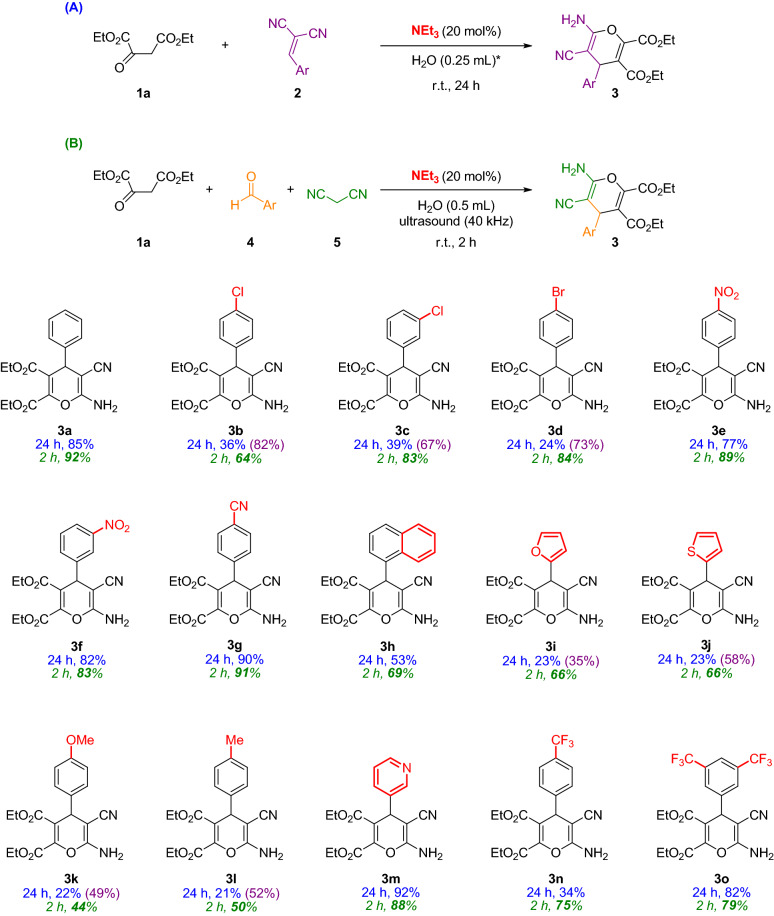
Catalytic routes followed to synthesize 4*H*-pyrans **3**. ^(*)^H_2_O:EtOH 5:1 (0.3 mL) if ethanol is needed. Scope of the catalytic syntheses of 4*H*-pyrans **3a**–**o**. Yields after column chromatography. Blue color: results following route A; Purple color (in brackets): results following route A and adding 50 μL EtOH; Green color (italics): results following route B.

After an extensive screening of the reaction conditions such as solvents, base catalysts, the concentration of the reagents and time, among others (see supporting information for more details, Tables S1 and S2), the scope of this process was explored for the synthesis of highly substituted 4*H*-pyrans **3**. Hence, in search of greener procedures, two new methods using pure water have been explored (Fig. 2, routes A and B). The first route involves the use of the preformed alkylidene malononitrile reagent **2**. Additionally, more interesting was the development of a multicomponent approach using ultrasounds, also carried out in pure water (Fig. 2B).

With the best reaction conditions in hand [Et_3_N (20 mol%) and H_2_O (0.25 mL), at room temperature (Table S1, entry 38)], final products **3** were obtained with good yields (up to 92%) following route A after 24 h (Fig. 2, yields in blue). The addition of 50 μL of EtOH in those cases where poor yields were obtained, rendered better results, maybe due to an improved solubility of all reagents in the reaction medium (Fig. 2, yields in purple). It is worth noting that the multicomponent approach performed in pure water at room temperature (Fig. 2B) gave rise to better results in shorter reaction times (2 h) (Fig. 2, yields in green). In this case, the use of ultrasounds as the activation way of the reaction was the key factor for the high yields obtained [not using ultrasounds under the same reaction conditions provides poorer yields (Table S2, entry 2)]. Moreover in this process, the bath temperature was not appreciably increased after 2 h. Therefore, it is expected that the reactions are activated by the ultrasound energy itself and no due to an increase in temperature of the reaction. In the multicomponent process, the reagents are used in equivalence as a clear example of atom economy^68^. The crudes of all these reactions are very clean and the purification and isolation of the products are carried out after a simple extraction from the same vessel and a fast column chromatography on silica gel.

Even though there is not a clear correlation between the substitution on the aromatic rings and the reactivity of the process following route A, it seems that for route B, electron-withdrawing groups in the aromatic ring render better yields in comparison with those bearing electron-donating groups or with heteroaromatic rings (Fig. 2).

**Figure 3:**
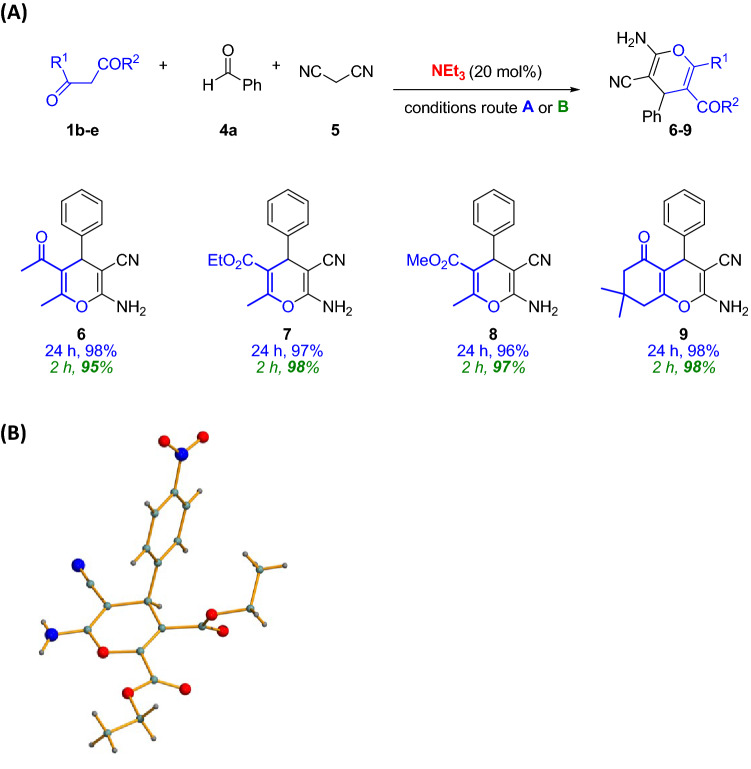
(**A**) Catalytic routes followed to synthesize 4*H*-pyrans **6–9**. Yields after column chromatography. Blue color: results following route A; Green color (italics): results following route B. (**B**) X-ray structure of 4*H*-pyran **3e**.

On further experiments, we were able to use other β-dicarbonyl compounds as nucleophiles (**1b–e**), giving rise to the desired products **6**–**9** also with excellent results using both routes in pure water (Fig. 3A). Interestingly, the multicomponent approach using ultrasounds allowed short reaction times.

Single crystal was grown from adduct **3e** and the structure was elucidated by X-ray diffraction. It shows the high functionalization of final target products (Fig. 3B)^69^.

### Biological activity: DNA interaction studies

In the last decade, there has been a growing interest in deepening the knowledge of drug interaction in the biological medium with the aim of understanding, among other aspects, the mechanism of action of active compounds. It is known that some small organic molecules, mostly planar ones, are able to stick to DNA and to disrupt the cellular cycle initiating programmed cell death. This aspect could be pivotal in some biological processes such as cancer, providing a rational design of new drugs and other strategies for cancer therapy^70–75^. Therefore, one of the most challenging goals in this area of research is the design and preparation of new small organic molecules able to bind to DNA with high selectivity and large association constants.

Based on our own experience in this field^76,77^, the plausible DNA interactions of 4*H*-pyrans synthesized in this work have been studied because of their wide range of biological properties^78,79^. Thus, the interaction effect of 4*H*-pyrans **3**,**6–9** towards calf thymus DNA (ctDNA) was investigated. For that purpose, firstly any possible kind of interaction between the compounds and ctDNA was studied. Then, the nature of the interaction was elucidated.

#### Calculation of the binding constant: UV–Vis spectra

There are several ways to calculate binding constants (*K*_b_) between drugs and DNA and, therefore, many techniques that allow to do so. Examples of these are fluorescence and absorption techniques^80–84^. We have selected UV–Vis because of its availability and straightforward handling. There are two procedures that can be used to perform the experiments. In the first one, the DNA is titrated with increasing amounts of the assayed compound. Then, the variations observed in the position and intensity of the DNA peak at 260 nm are measured and the data processed to obtain binding constants and hints about the plausible interaction modes^85^. However, this method presents a limitation. The small extinction coefficient of the DNA leads to a worse precision in the calculation of the resulting binding constant. In contrast, if the experiment is conducted taking as reference the peaks of the studied compounds, usually with stronger absorptions, bigger changes in the bands can be observed. Thus, better precisions on the *K*_b_ are expected to be obtained^86,87^. Therefore, the second methodology was selected to elucidate the *K*_b_ of compounds **3a**–**3o**, **6**–**9** with ctDNA. An example of the titration experiments can be seen in Fig. S33 for compound **3n**^88–90^.

In this case, **3n** showed two intensive absorption bands at 242 and 295 nm, which are typically associated with π → π* and n → π* electronic transitions, respectively. The successive additions of DNA promoted a hypochromic effect in the peak at 295 nm and a hyperchromic effect in the peak at 242 nm, indicative of an interaction between compound **3n** and ctDNA. Similarly, compounds **3** and **6**–**9** also showed diverse variations of the intensity of their absorption bands to different degrees, after subsequent addition of ctDNA, see Fig. S20–38. The binding constant was then calculated for all of them using the modified Benesi-Hildebrand equation (see Fig. S39 for an example)^91–93^.

**Figure 4:**
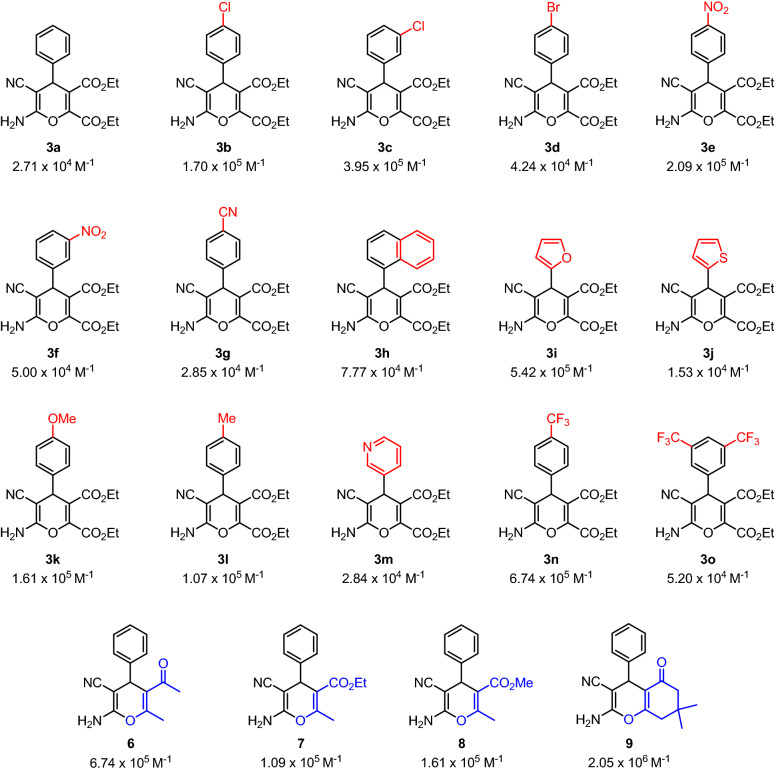
Binding constants (*K*_b_) obtained for compounds **3a–o**, **6–9**.

*K*_b_ values range from 1.53 × 10^4^ M^−1^ to 2.05 × 10^6^ M^−1^, being the majority of them of the order of 10^5^ M^−1^. In Fig. 4, a summary of the binding constants obtained for every compound of this work is reported. The calculated *K*_b_ evidence a high affinity of the new 4*H*-pyrans for ctDNA base pairs. The highest *K*_b_value presented by **9** (2.05 × 10^6^ M^−1^) indicates a strong binding towards ctDNA. It is noteworthy that the binding constant for ethidium bromide (EtBr)^94^, a well-known intercalative agent, is 1.37 × 10^5^ M^−1^^95^, suggests that these 4*H*-pyrans could have similar interaction with DNA. It is also known that typical *K*_b_ values for intercalative compounds range from 10^4^ to 10^6^ M^−1^, whereas for groove binders are between 10^5^ M^−1^ to 10^9^ M^−1^^96,97^. Therefore, further experiments were performed to elucidate the interaction mode with DNA. Additionally, a closer look at the *K*_b_ values does not show a straightforward relationship between the strength of the interaction with ctDNA and the electronic properties or structure–property relationships of the products, which might indicate that the pyran core is the main responsible of such interaction. These high binding affinities could be due to the presence of the esters and the NH_2_ groups in the pyran skeletons, which are able to establish additional interactions and hydrogen bonding forces with the base pairs of DNA molecule^78^.

#### Determination of the DNA binding type

The binding modes of a drug or a small organic molecule to DNA could be categorized into^98^: (1) a strong covalent union such as that exhibited by cisplatin^99^; or (2) weaker unions through intermolecular forces^100^ (such as van der Waals, hydrogen bonding, π stacking, etc.) in which intercalative molecules can be found (e.g. ethidium bromide)^101–103^ and groove binding ones. Groove bindings are categorized into two subclasses, minor and major groove binding. Such variation refers to the differences found in the grooves of the macrostructure of DNA. Finally, (3) weakest union to DNA is driven by electrostatic interactions between the drug (or the studied molecule, in general) and the phosphorated scaffold of the double-strand. Many experiments could bring light upon the binding mechanism^104^, such as the study of the viscosity, circular dichroism or fluorescence quenching, among others. We have analyzed these properties in this work to shed light on the DNA binding type of our synthesized compounds.

##### Viscosity measurements

A very simple technique, such as viscometry, can provide a lot of information. It is considered as one of the best methods for studies in solution because of its high sensibility towards changes in the hydrodynamic properties of the DNA^105–107^. In these experiments, an increase of the viscosity is observed when an intercalative compound is measured. DNA length tends to increase due to the higher base pairs separation promoted by the intercalative molecule inserted between them. In contrast, when a compound establishes covalent bonds with the DNA, its structure tends to bend and this leads to an average reduction of its length, causing a decrease of the viscosity. Any other interaction does not cause any significant influence^108–110^.

In the present work, the experiment is conducted with compound **3n** as a model molecule. **3n** has been selected because it shows one of the highest calculated binding constants (see Fig. 4) facilitating information gathering. Moreover, its structural similarity with the other compounds will allow an easy extrapolation of the results obtained in these studies. Specifically, ctDNA was placed in a thermostatic bath at 298 K with a Cannon–Fenske viscometer and successive additions of **3n** were performed. In Fig. S40, a plot of ƞ/ƞ_0_ *vs* the ratio of **3n** to DNA concentration is presented. The values depicted in Fig. S40 support that the successive additions of **3n** to the solution of ctDNA resulted in no significant change in the relative viscosity of the whole mixture. This finding might indicate that **3n** interacts with ctDNA with either minor or major groove binding, discarding the intercalative hypothesis.

##### Circular dichroism (CD)

**Figure 5:**
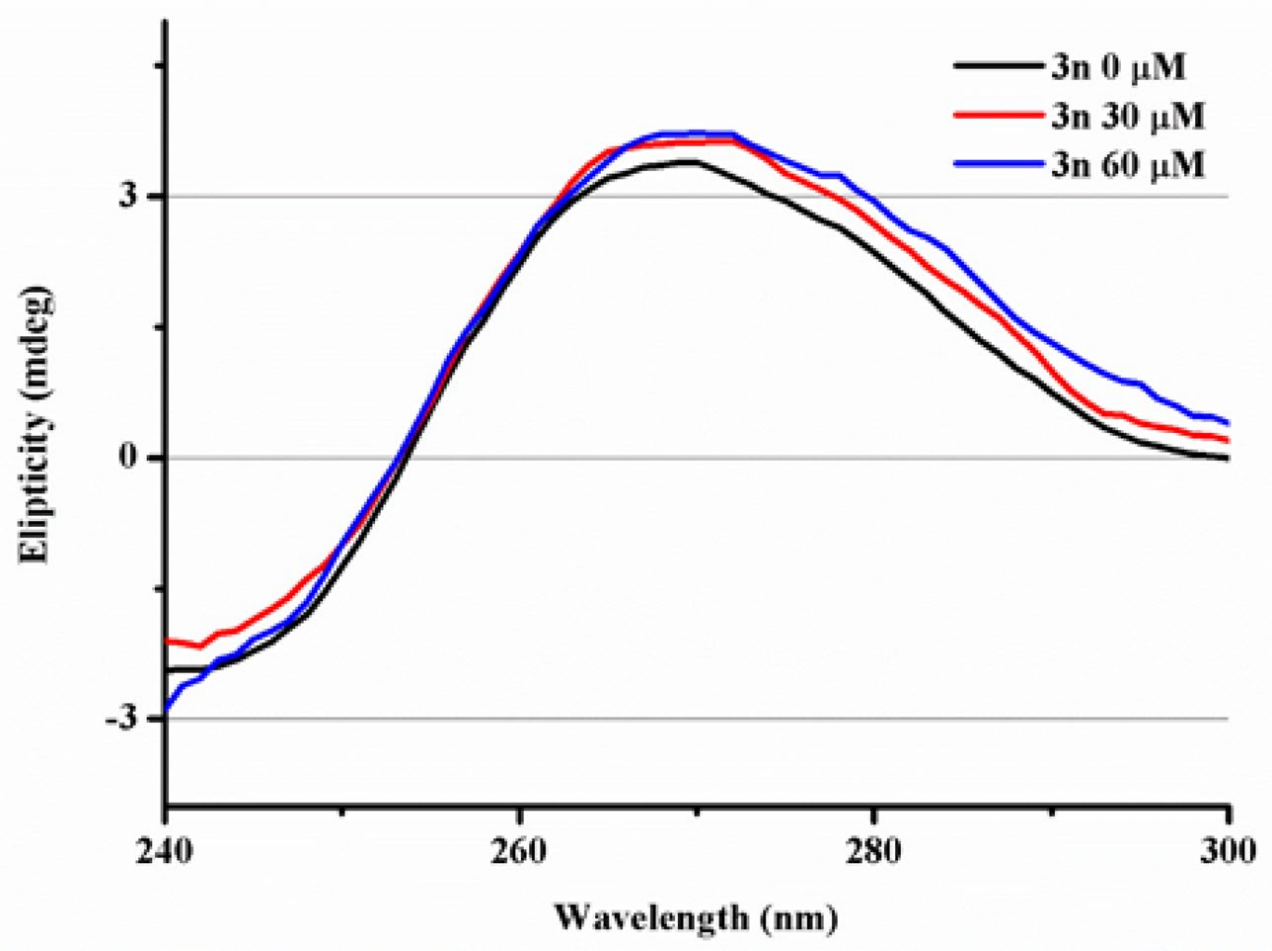
CD spectra of ctDNA (28 µM) in a buffer solution of Tris/HCl (0.1 M, pH 7.2), with a baseline correction of the buffer with a 1 cm path length at 298 K. Two additional experiments are recorded adding compound **3n** at 30 and 60 µM over a solution of ctDNA.

This is a very sensitive technique that allows to see tiny changes in the secondary structure of DNA upon binding with a drug or a small organic molecule^111^. In the circular dichroism spectra of ctDNA, two bands can be seen at 243 (negative) and 277 (positive) nm caused by the helicity and base stacking, respectively. These bands are very sensitive towards binding molecules^112^. ctDNA spectra show no changes or small changes when a minor/major groove intercalation or electrostatic binding takes place. However, when an intercalative molecule is examined both bands should suffer considerable changes^113–115^. In Fig. 5, the experiments of CD conducted with compound **3n** can be analyzed.

In this case, the band at 243 nm does not give information due to the distortion caused upon addition of the increasing concentrations of compound **3n**. Interestingly, the positive band at 277 nm shows no apparent changes, which is not compatible with an intercalation binding. This experiment supports the hypothesis raised from the viscosity experiment, suggesting a minor/major groove binding mode. Thereby, a more specific experiment is required to discern between both kinds of bindings.

##### Competitive assays of fluorescence quenching

**Figure 6:**
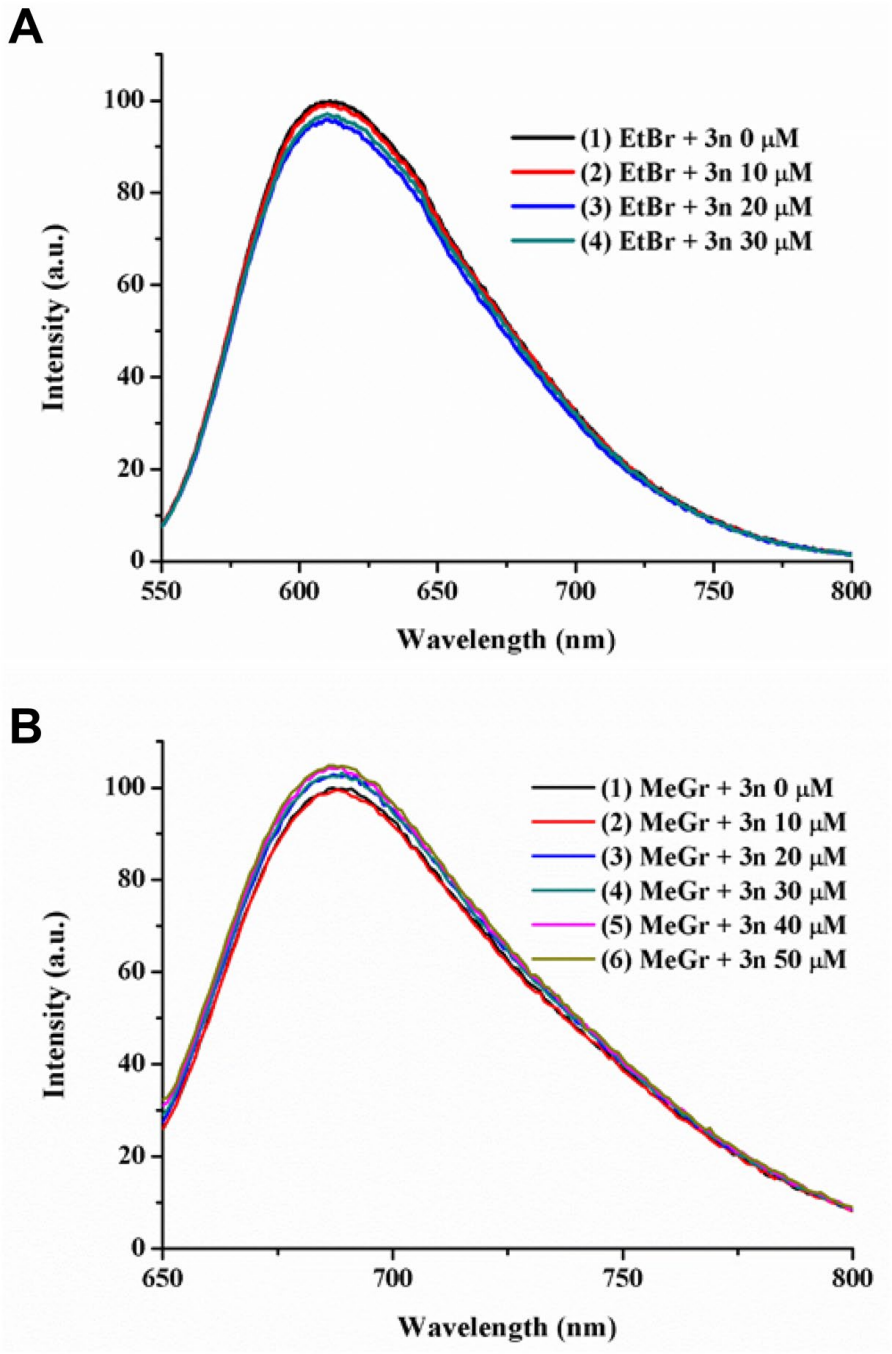
(**A**) Titration experiment of a solution of ctDNA (50 µM) and ethidium bromide (2.5 µM) in Tris/HCl (0.1 M, pH 7.2) with increasing concentrations of compound **3n** (0–30 µM). (**B**) Titration experiment of a solution of ctDNA (50 µM) and methyl green (2.5 µM) in Tris/HCl (0.1 M, pH 7.2) with increasing concentrations of compound **3n** (0–50 µM). Both plots are normalized to the maximum intensity of the initial experiment.

**Figure 7:**
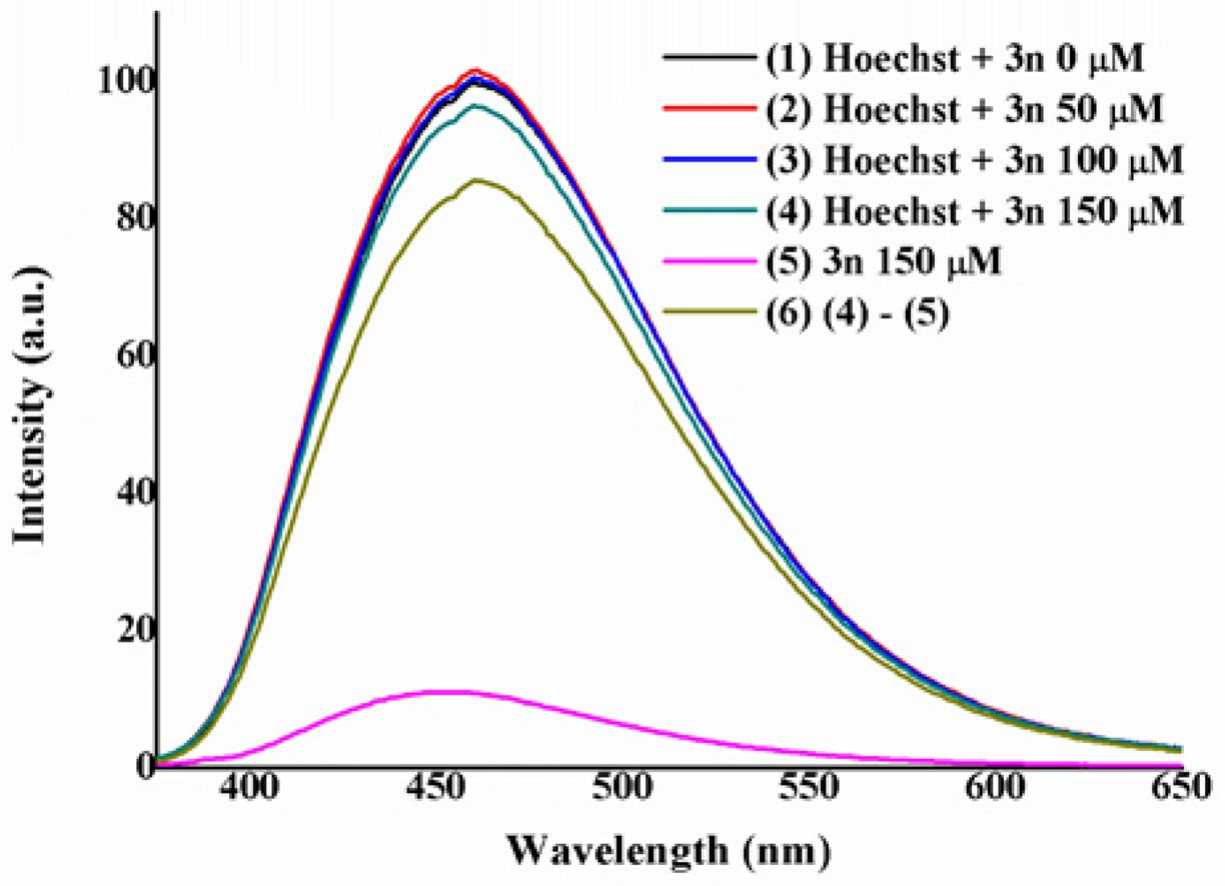
Titration experiment of a solution of ctDNA (200 µM) and Hoechst 33342 (2.5 µM) in Tris/HCl (0.1 M, pH 7.2) with increasing concentrations of compound **3n** (0–150 µM). The plot is normalized to the maximum intensity of the initial experiment.

In order finally to elucidate the binding mode of 4*H*-pyran **3n** with ctDNA, three experiments of fluorescence quenching have been performed. Previous studies abovementioned have shown that, most likely, **3n** and by structural analogy compounds **3a–3o** and **6–9** bind to the minor/major groove of the DNA. Therefore, three commercially available luminescent model compounds such as ethidium bromide (EtBr, an intercalator)^116,117^, methyl green (MeGr, major groove binder)^118,119^ and Hoechst 33342 (minor groove binder) have been used to assess the type of interactions established^120–123^. The reasoning behind this experiment is that when 4*H*-pyrans are added to a mixture of ctDNA and the model molecule (specially selected because of its strong emission), the fluorescence drops only when both compounds compete for the same binding position of the DNA. Higher concentrations of 4*H*-pyrans than those of the model molecules are used to grant their substitution from the DNA. Figures 6 and 7 show the results obtained in this study against the three model compounds.

In Fig. 6A, a slight decrease in the maximum intensity of EtBr takes place after the subsequent addition of compound **3n**. This could indicate that there is a small interaction. However, previous experiments exclude such possibility and due to the slight change observed on the emission, it can be overseen. In the case of Fig. 6B, no quenching of the emission is detected when using MeGr. These results would discard a plausible major groove binding of the ctDNA and **3n**. Similarly, the quenching experiment performed using Hoechst 33342 as a model molecule showed little diminution of the maximum emission intensity of the dye (Fig. 7).

Therefore, it seems that none of the three competition experiments showed the displacement of the model molecule (EtBr, MeGr or Hoechst) by the pyran derivative. However, it is known that emission from the synthesized pyran derivatives in Tris/HCl (0.1 M, pH 7.2) lies between 400 and 550 nm with a maximum intensity c.a. 455 nm [see plot (5) in Fig. 7]. Hence, emission from **3n** could be masking the competition experiment of Hoechst, as both of them are excited and emit in the same area of the spectrum. Figure S41 showed the emission maxima of **3n** and those of EtBr, MeGr and Hoechst, demonstrating that only the emission of Hoechst is affected by the presence of **3n**. Consequently, after appropriate corrections on the Hoechst competition experiment [see plots (4), (5) and (6) in Fig. 7] a significant drop in the intensity for the emission of Hoechst was observed [see Fig. 7, plot (6)]. Such a decrease of the emission intensity suggests that **3n** was displacing Hoechst from the minor groove of DNA^124^.

**Figure 8:**
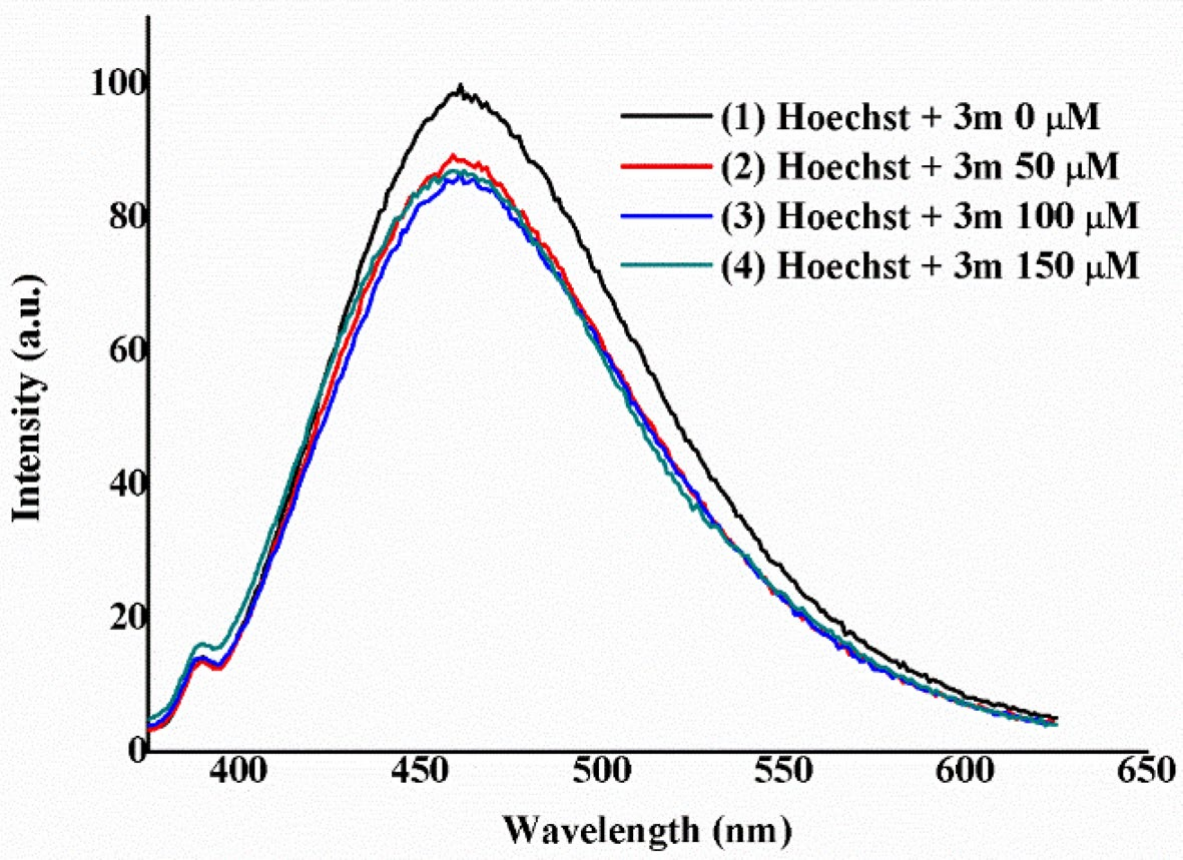
Titration experiment of a solution of ctDNA (200 µM) and Hoechst 33342 (2.5 µM) in Tris/HCl (0.1 M, pH 7.2) with increasing concentrations of compound **3m** (0–150 µM). The plot is normalized to the maximum intensity of the initial experiment.

A new competition experiment was then designed considering a different pyran derivative, in order to extrapolate the behavior observed with compound **3n** to their analogs. Thus, compound **3m** was also examined as a possible minor groove binder using a competitive fluorescence experiment with Hoechst (Fig. 8). Similarly, quenching of the emission was observed after the addition of **3m** to the mixture of ctDNA and Hoechst, demonstrating once again that these pyrans are minor groove binders.

As a summary, the high binding constants obtained from UV–Vis indicate that the synthesized pyran derivatives strongly interact with ctDNA, opening the scope to distinguish between intercalation or minor/major groove bindings. CD and viscometry show results not compatible with intercalation, whereas the fluorescence quenching profiles suggest a minor groove interaction as a plausible binding mode.

## Conclusion

A very powerful and sustainable multicomponent approach for the synthesis of new highly substituted 4*H*-pyran derivatives is described. Two different protocols using accessible and efficient Et_3_N as a simple catalyst in a 20 mol% in water have been developed. The use of the multicomponent approach for this process with ultrasounds affords excellent results, for the first time^125^. The extremely simple operational methodology, short reaction times, clean procedure and high product yields render this new protocol highly appealing for the synthesis of 4*H*-pyran derivatives, with high potential as therapeutic agents. DNA binding studies of the final products have been performed by viscosity measurements, circular dichroism, UV–visible absorption and fluorescence spectroscopy. These studies allow to conclude that the synthesized 4*H-*pyrans bind to DNA through the minor groove rather than to major groove or by intercalation, with a higher *K*_b_ than those previously reported for a 4*H*-pyran^78,79^. This work represents one of the scarce studies carried out with pyrans and their DNA binding interactions, thus opening the door for the future development of these scaffolds as promising drugs.

## Experimental section

### General experimental methods and instrumentation^48^

Purification of reaction products was carried out by column chromatography using silica gel (0.063–0.200 mm). Analytical thin-layer chromatography was performed on 0.25 mm silica gel 60-F plates. ESI ionization method and mass analyzer type MicroTof-Q were used for the HRMS measurements. NMR spectra were recorded at room temperature on a Bruker ARX300 or AV400 instruments. ^1^H-NMR spectra were recorded at 300 or 400 MHz, and ^13^C-APT-NMR spectra were recorded at 75 or 100 MHz, using DMSO-*d*_6_ as the deuterated solvent. Chemical shifts were reported in the *δ* scale relative to residual DMSO (2.50 ppm) for ^1^H-NMR and to the central line of DMSO-*d*_6_ (39.43 ppm) for ^13^C-APT-NMR. A Branson 5510 ultrasonic bath is used in the synthesis of the final compounds. Melting points were determined on a Gallenkamp variable heating apparatus. IR spectra were recorded on a PerkinElmer FT-IR 2,400 microanalyzer.

All commercially available solvents and reagents were used as received.

The Softwares used to perform the Figures are ChemBioDraw Ultra 9.0, Origin Pro 9.0 and Powerpoint 2010.

### General procedures for the synthesis of 4*H*-pyran derivatives 3a–o, 6–9

Route (A): To a mixture of the corresponding benzylidenemalononitrile **2** (0.1 mmol) and triethylamine (20 mol%, 2.8 μL) in water (0.25 mL; and 50 μL of EtOH, if needed), enol derivative **1** (0.2 mmol) was added. The reaction mixture was stirred at room temperature and monitored by TLC (*n-*hexane:ethyl acetate 7:3) until the total consumption of benzylidenemalononitrile **2**.

Route (B): To a mixture of 0.5 mL of a stock solution in H_2_O of malononitrile **5** (0.1 mmol), triethylamine (20 mol%, 2.8 μL) and the corresponding aldehyde **4** (0.1 mmol), enol derivative **1** (0.1 mmol) was added. The reaction mixture was then introduced in an ultrasonic bath (40 kHz). The reaction mixture was monitored by TLC (*n-*hexane:ethyl acetate 7:3) until the total consumption of aldehyde **4**.

### Characterization of 4*H*-pyran derivatives 3a–o, 6–9

#### Diethyl 6-amino-5-cyano-4-phenyl-4*H*-pyran-2,3-dicarboxylate (**3a**)

Following the general procedures, compound **3a** was obtained as a white solid in 85% yield (29.1 mg), after 24 h of reaction at room temperature (route A); and in 92% yield (31.5 mg), after 2 h of reaction at room temperature (route B). The purification was performed by extraction with EtOAc (3 × 0.25 mL) and the column chromatography with *n-*hexane:ethyl acetate from 8:2 to 6:4. M.p. 137–139 °C. ^1^H-NMR (300 MHz, DMSO-*d*_6_) *δ* 0.98 (t, *J* = 7.1 Hz, 3H), 1.25 (t, *J* = 7.1 Hz, 3H), 3.97 (q, *J* = 7.1 Hz, 2H), 4.21–4.31 (m, 2H), 4.39 (s, 1H), 7.16–7.30 (m, 5H), 7.33–7.39 (m, 2H). ^13^C-APT-NMR (75 MHz, DMSO-*d*_6_) *δ* 13.5 (1C), 13.7 (1C), 39.0 (1C), 56.5 (1C), 61.2 (1C), 62.5 (1C), 113.3 (1C), 119.1 (1C), 127.5 (1C), 127.6 (2C), 128.7 (2C), 142.5 (1C), 143.7 (1C), 158.5 (1C), 160.6 (1C), 164.0 (1C). IR (neat) (cm^−1^) ν 3,409, 3,328, 3,269, 3,226, 3,199, 2,981, 2,938, 2,200, 2,159, 2,029, 1,977, 1,752, 1,711, 1,673, 1,650, 1,605, 1,197, 1,106, 1,047, 736, 698, 421. HRMS (ESI+) calcd for C_18_H_18_N_2_NaO_5_ 365.1108; found 365.1116 [M + Na].

#### Diethyl 6-amino-4-(4-chlorophenyl)-5-cyano-4*H*-pyran-2,3-dicarboxylate (**3b**)

Following the general procedures, compound **3b** was obtained as a white solid in 36% yield (13.6 mg), 82% yield (30.9 mg) if 50 µL of EtOH are added, after 24 h of reaction at room temperature (route A); and in 64% yield (24.1 mg), after 2 h of reaction at room temperature (route B). The purification was performed by extraction with EtOAc (3 × 0.25 mL) and the column chromatography with *n-*hexane:ethyl acetate from 8:2 to 6:4. M.p. 108–110 °C. ^1^H-NMR (300 MHz, DMSO-*d*_6_) *δ* 1.00 (t, *J* = 7.1 Hz, 3H), 1.25 (t, *J* = 7.1 Hz, 3H), 3.98 (q, *J* = 7.1 Hz, 2H), 4.21–4.31 (m, 2H), 4.45 (s, 1H), 7.19–7.23 (m, 2H), 7.27 (bs, 2H), 7.41–7.45 (m, 2H). ^13^C-APT-NMR (100 MHz, DMSO-*d*_6_) *δ* 13.5 (1C), 13.6 (1C), 38.3 (1C), 56.1 (1C), 61.2 (1C), 62.5 (1C), 112.8 (1C), 118.9 (1C), 128.7 (2C), 129.5 (2C), 132.1 (1C), 141.5 (1C), 143.8 (1C), 158.5 (1C), 160.4 (1C), 163.8 (1C). IR (neat) (cm^−1^) ν 3,419, 3,340, 3,279, 3,229, 3,200, 2,982, 2,971, 2,200, 2,024, 1,977, 1,731, 1,715, 1,686, 1,649, 1,605, 1,413, 1,370, 1,338, 1,290, 1,261, 1,190, 1,172, 1,087, 1,042, 1,010, 822, 776, 445. HRMS (ESI+) calcd for C_18_H_17_ClN_2_NaO_5_ 399.0718; found 399.0737 [M + Na].

#### Diethyl 6-amino-4-(3-chlorophenyl)-5-cyano-4*H*-pyran-2,3-dicarboxylate (**3c**)

Following the general procedures, compound **3c** was obtained as a white solid in 39% yield (14.7 mg), 67% yield (25.2 mg) if 50 µL of EtOH are added, after 24 h of reaction at room temperature (route A); and in 83% yield (31.3 mg), after 2 h of reaction at room temperature (route B). The purification was performed by extraction with EtOAc (3 × 0.25 mL) and the column chromatography with *n-*hexane:ethyl acetate from 8:2 to 6:4. M.p. 127–129 °C. ^1^H-NMR (400 MHz, DMSO-*d*_6_) *δ* 1.00 (t, *J* = 7.1 Hz, 3H), 1.25 (t, *J* = 7.1 Hz, 3H), 3.96–4.03 (m, 2H), 4.23–4.30 (m, 2H), 4.48 (s, 1H), 7.15–7.18 (m, 1H), 7.22–7.23 (m, 1H), 7.27 (bs, 2H), 7.34–7.37 (m, 1H), 7.39–7.43 (m, 1H). ^13^C-APT-NMR (100 MHz, DMSO-*d*_6_) *δ* 13.5 (1C), 13.6 (1C), 38.5 (1C), 55.9 (1C), 61.2 (1C), 62.5 (1C), 112.7 (1C), 118.8 (1C), 126.4 (1C), 127.5 (1C), 127.5 (1C), 130.7 (1C), 133.1 (1C), 143.9 (1C), 145.0 (1C), 158.5 (1C), 160.4 (1C), 163.8 (1C). IR (neat) (cm^−1^) ν 3,417, 3,327, 3,264, 3,224, 3,197, 2,985, 2,198, 2,160, 2,035, 1,738, 1,711, 1,671, 1,604, 1,254, 1,203, 1,106, 1,044, 794, 749, 660, 414. HRMS (ESI+) calcd for C_18_H_17_ClN_2_NaO_5_ 399.0718; found 399.0732 [M + Na].

#### Diethyl 6-amino-4-(4-bromophenyl)-5-cyano-4*H*-pyran-2,3-dicarboxylate (**3d**)

Following the general procedures, compound **3d** was obtained as a white solid in 24% yield (10.1 mg), 73% yield (30.8 mg) if 50 µL of EtOH are added, after 24 h of reaction at room temperature (route A); and in 84% yield (35.4 mg), after 2 h of reaction at room temperature (route B). The purification was performed by extraction with EtOAc (3 × 0.25 mL) and the column chromatography with *n-*hexane:ethyl acetate from 8:2 to 6:4. M.p. 111–113 °C. ^1^H-NMR (400 MHz, DMSO-*d*_6_) *δ* 1.01 (t, *J* = 7.1 Hz, 3H), 1.25 (t, *J* = 7.1 Hz, 3H), 3.95–4.02 (m, 2H), 4.22–4.30 (m, 2H), 4.43 (s, 1H), 7.13–7.17 (m, 2H), 7.25 (bs, 2H), 7.55–7.58 (m, 2H). ^13^C-APT-NMR (100 MHz, DMSO-*d*_6_) *δ* 13.5 (1C), 13.6 (1C), 38.4 (1C), 56.0 (1C), 61.2 (1C), 62.5 (1C), 112.7 (1C), 118.8 (1C), 120.6 (1C), 129.8 (2C), 131.6 (2C), 141.9 (1C), 143.9 (1C), 158.5 (1C), 160.4 (1C), 163.8 (1C). IR (neat) (cm^−1^) ν 3,419, 3,339, 3,276, 3,229, 3,199, 2,970, 2,199, 2,160, 2,028, 1,977, 1,732, 1,714, 1,686, 1,649, 1,604, 1,415, 1,370, 1,338, 1,289, 1,262, 1,189, 1,173, 1,089, 1,041, 1,010, 820, 775, 454. HRMS (ESI+) calcd for C_18_H_17_BrN_2_NaO_5_ 443.0213; found 443.0220 [M + Na].

#### Diethyl 6-amino-5-cyano-4-(4-nitrophenyl)-4*H*-pyran-2,3-dicarboxylate (**3e**)

Following the general procedures, compound **3e** was obtained as a white solid in 77% yield (29.8 mg), after 24 h of reaction at room temperature (route A); and in 89% yield (34.5 mg), after 2 h of reaction at room temperature (route B). The purification was performed by extraction with EtOAc (3 × 0.25 mL) and the column chromatography with *n-*hexane:ethyl acetate from 8:2 to 6:4. M.p. 103–105 °C. ^1^H-NMR (400 MHz, DMSO-*d*_6_) *δ* 1.00 (t, *J* = 7.1 Hz, 3H), 1.26 (t, *J* = 7.1 Hz, 3H), 3.98 (q, *J* = 7.1 Hz, 2H), 4.28 (q, *J* = 7.1 Hz, 2H), 4.64 (s, 1H), 7.36 (bs, 2H), 7.48–7.51 (m, 2H), 8.23–8.26 (m, 2H). ^13^C-APT-NMR (100 MHz, DMSO-*d*_6_) *δ* 13.5 (1C), 13.6 (1C), 38.5 (1C), 55.5 (1C), 61.3 (1C), 62.6 (1C), 111.6 (1C), 118.6 (1C), 124.0 (2C), 129.0 (2C), 144.8 (1C), 146.8 (1C), 150.0 (1C), 158.5 (1C), 160.4 (1C), 163.5 (1C). IR (neat) (cm^−1^) ν 3,418, 3,340, 3,276, 3,226, 3,199, 2,979, 2,934, 2,329, 2,199, 2,118, 1,998, 1,729, 1,712, 1,687, 1,649, 1,604, 1,524, 1,350, 1,338, 1,291, 1,263, 1,195, 1,173, 1,091, 1,045, 1,013, 819, 777, 451. HRMS (ESI+) calcd for C_18_H_17_N_3_NaO_7_ 410.0959; found 410.0976 [M + Na].

#### Diethyl 6-amino-5-cyano-4-(3-nitrophenyl)-4*H*-pyran-2,3-dicarboxylate (**3f**)

Following the general procedures, compound **3f** was obtained as a white solid in 82% yield (31.8 mg), after 24 h of reaction at room temperature (route A); and in 83% yield (32.1 mg), after 2 h of reaction at room temperature (route B). The purification was performed by extraction with EtOAc (3 × 0.25 mL) and the column chromatography with *n-*hexane:ethyl acetate from 8:2 to 6:4. M.p. 154–156ºC. ^1^H-NMR (400 MHz, DMSO-*d*_6_) *δ* 0.99 (t, *J* = 7.1 Hz, 3H), 1.25 (t, *J* = 7.1 Hz, 3H), 3.98 (q, *J* = 7.1 Hz, 2H), 4.27 (q, *J* = 7.1 Hz, 2H), 4.71 (s, 1H), 7.36 (bs, 2H), 7.68–7.72 (m, 2H), 8.04–8.05 (m, 1H), 8.15–8.20 (m, 1H). ^13^C-APT-NMR (100 MHz, DMSO-*d*_6_) *δ* 13.4 (1C), 13.6 (1C), 38.3 (1C), 55.6 (1C), 61.3 (1C), 62.6 (1C), 112.1 (1C), 118.7 (1C), 122.2 (1C), 122.6 (1C), 130.5 (1C), 134.5 (1C), 144.5 (1C), 144.9 (1C), 147.8 (1C), 158.7 (1C), 160.4 (1C), 163.6 (1C). IR (neat) (cm^−1^) ν 3,398, 3,330, 3,267, 3,225, 3,201, 3,087, 2,983, 2,199, 2,159, 3,032, 1,725, 1,703, 1,674, 1,649, 1,606, 1,530, 1,346, 1,306, 1,260, 1,197, 1,170, 1,101, 1,026, 825, 736, 506. HRMS (ESI+) calcd for C_18_H_17_N_3_NaO_7_ 410.0959; found 410.0966 [M + Na].

#### Diethyl 6-amino-5-cyano-4-(4-cyanophenyl)-4*H*-pyran-2,3-dicarboxylate (**3g**)

Following the general procedures, compound **3g** was obtained as a white solid in 90% yield (33.1 mg), after 24 h of reaction at room temperature (route A); and in 91% yield (33.4 mg), after 2 h of reaction at room temperature (route B). The purification was performed by extraction with EtOAc (3 × 0.25 mL) and the column chromatography with *n-*hexane:ethyl acetate from 8:2 to 6:4. M.p. 103–105 °C. ^1^H-NMR (300 MHz, DMSO-*d*_6_) *δ* 0.99 (t, *J* = 7.1 Hz, 3H), 1.25 (t, *J* = 7.1 Hz, 3H), 3.98 (q, *J* = 7.1 Hz, 2H), 4.27 (q, *J* = 7.1 Hz, 2H), 4.56 (s, 1H), 7.35 (bs, 2H), 7.39–7.41 (m, 2H), 7.84–7.87 (m, 2H). ^13^C-APT-NMR (100 MHz, DMSO-*d*_6_) *δ* 13.5 (1C), 13.6 (1C), 38.7 (1C), 55.6 (1C), 61.3 (1C), 62.5 (1C), 110.3 (1C), 111.8 (1C), 118.6 (1C), 118.7 (1C), 128.7 (2C), 132.7 (2C), 144.6 (1C), 148.1 (1C), 158.5 (1C), 160.4 (1C), 163.6 (1C). IR (neat) (cm^−1^) ν 3,333, 3,186, 2,922, 2,852, 2,231, 2,196, 2,122, 1,993, 1,718, 1,679, 1,641, 1,603, 1,370, 1,297, 1,251. 1,192, 1,096, 1,017, 846, 557. HRMS (ESI+) calcd for C_19_H_17_N_3_NaO_5_ 390.1060; found 390.1066 [M + Na].

#### Diethyl 6-amino-5-cyano-4-(naphthalen-1-yl)-4*H*-pyran-2,3-dicarboxylate (**3h**)

Following the general procedures, compound **3h** was obtained as a white solid in 53% yield (20.8 mg), after 24 h of reaction at room temperature (route A); and in 69% yield (27.1 mg), after 2 h of reaction at room temperature (route B). The purification was performed by extraction with EtOAc (3 × 0.25 mL) and the column chromatography with *n-*hexane:ethyl acetate from 8:2 to 6:4. M.p. 117–119 °C. ^1^H-NMR (300 MHz, DMSO-*d*_6_) *δ* 0.65 (t, *J* = 7.1 Hz, 3H), 1.26 (t, *J* = 7.1 Hz, 3H), 3.70–3.80 (m, 2H), 4.22–4.32 (m, 2H), 5.41 (s, 1H), 7.18 (bs, 2H), 7.34 (dd, *J* = 7.2, 1.1 Hz, 1H), 7.52–7.61 (m, 3H), 7.86 (d, *J* = 8.2 Hz, 1H), 7.94–7.97 (m, 1H), 8.29 (d, *J* = 8.1 Hz, 1H). ^13^C-APT-NMR (100 MHz, DMSO-*d*_6_) *δ* 13.1 (1C), 13.6 (1C), 57.0 (1C), 60.9 (1C), 62.4 (1C), 114.2 (1C), 119.0 (1C), 123.1 (1C), 125.8 (1C), 125.8 (1C), 126.2 (1C), 126.5 (1C), 127.9 (1C), 128.5 (1C), 130.8 (1C), 133.3 (1C), 158.4 (1C), 160.5 (1C), 164.0 (1C). IR (neat) (cm^−1^) ν 3,405, 3,326, 3,269, 3,224, 3,201, 2,958, 2,923, 2,852, 2,198, 2,160, 2,023, 1,976, 1,753, 1,714, 1,674, 1,646, 1,608, 1,291, 1,252, 1,185, 1,160, 1,098, 1,040, 1,003, 863, 775, 447. HRMS (ESI+) calcd for C_22_H_20_N_2_NaO_5_ 415.1264; found 415.1261 [M + Na].

#### Diethyl 6-amino-5-cyano-4-(furan-2-yl)-4*H*-pyran-2,3-dicarboxylate (**3i**)

Following the general procedures, compound **3i** was obtained as a white solid in 23% yield (7.6 mg), 35% yield (11.6 mg) if 50 µL of EtOH are added, after 24 h of reaction at room temperature (route A); and in 66% yield (21.9 mg), after 2 h of reaction at room temperature (route B). The purification was performed by extraction with EtOAc (3 × 0.25 mL) and the column chromatography with *n-*hexane:ethyl acetate from 8:2 to 6:4. M.p. 92–94 °C. ^1^H-NMR (300 MHz, DMSO-*d*_6_) *δ* 1.09 (t, *J* = 7.1 Hz, 3H), 1.25 (t, *J* = 7.1 Hz, 3H), 4.06 (q, *J* = 7.1, Hz, 2H), 4.22–4.30 (m, 2H), 4.56 (s, 1H), 6.20–6.21–6.23 (m, 1H), 6.39 (dd, *J* = 3.2, 1.9 Hz, 1H), 7.27 (bs, 2H), 7.59 (dd, *J* = 1.9, 0.9 Hz, 1H). ^13^C-APT-NMR (100 MHz, DMSO-*d*_6_) *δ* 13.6 (1C), 13.6 (1C), 32.6 (1C), 53.7 (1C), 61.3 (1C), 62.5 (1C), 106.8 (1C), 110.6 (1C), 110.8 (1C), 118.8 (1C), 142.9 (1C), 144.6 (1C), 153.5 (1C), 159.2 (1C), 160.4 (1C), 163.8 (1C). IR (neat) (cm^−1^) ν 3,146, 2,124, 3,042, 2,986, 2,923, 2,224, 2,091, 1,991, 1,739, 1,605, 1,527, 1,456, 1,394, 1,296, 1,018, 934, 763, 583, 458. HRMS (ESI+) calcd for C_16_H_16_N_2_NaO_6_ 355.0901; found 355.0918 [M + Na].

#### Diethyl 6-amino-5-cyano-4-(thiophen-2-yl)-4*H*-pyran-2,3-dicarboxylate (**3j**)

Following the general procedures, compound **3j** was obtained as a white solid in 23% yield (8.0 mg), 58% yield (20.2 mg) if 50 µL of EtOH are added, after 24 h of reaction at room temperature (route A); and in 66% yield (23.0 mg), after 2 h of reaction at room temperature (route B). The purification was performed by extraction with EtOAc (3 × 0.25 mL) and the column chromatography with *n-*hexane:ethyl acetate from 8:2 to 6:4. M.p. 97–99 °C. ^1^H-NMR (300 MHz, DMSO-*d*_6_) *δ* 1.09 (t, *J* = 7.1 Hz, 3H), 1.25 (t, *J* = 7.1 Hz, 3H), 4.03–4.10 (m, 2H), 4.22–4.29 (m, 2H), 4.76 (s, 1H), 6.91–6.92 (m, 1H), 6.97 (dd, *J* = 5.1, 3.5 Hz, 1H), 7.30 (bs, 2H), 7.45 (dd, *J* = 5.1, 1.2 Hz, 1H). ^13^C-APT-NMR (100 MHz, DMSO-*d*_6_) *δ* 13.6 (1C), 13.6 (1C), 33.8 (1C), 56.6 (1C), 61.3 (1C), 62.5 (1C), 112.7 (1C), 118.9 (1C), 125.2 (1C), 125.8 (1C), 127.0 (1C), 144.0 (1C), 146.9 (1C), 158.7 (1C), 160.5 (1C), 163.7 (1C). IR (neat) (cm^−1^) ν 3,411, 3,329, 3,267, 3,225, 3,199, 2,982, 2,936, 2,200, 2,160, 2,033, 1,978, 1,747, 1,708, 1,674, 1,650, 16,051,370, 1,250, 1,196, 1,170, 1,101, 1,044, 1,009, 855, 697, 417. HRMS (ESI+) calcd for C_16_H_16_N_2_NaO_5_S 371.0672; found 371.0681 [M + Na].

#### Diethyl 6-amino-5-cyano-4-(4-methoxyphenyl)-4**H**-pyran-2,3-dicarboxylate (**3k**)

Following the general procedures, compound **3k** was obtained as a white solid in 22% yield (8.2 mg), 49% yield (18.2 mg) if 50 µL of EtOH are added, after 24 h of reaction at room temperature (route A); and in 44% yield (16.4 mg), after 2 h of reaction at room temperature (route B). The purification was performed by extraction with EtOAc (3 × 0.25 mL) and the column chromatography with *n-*hexane:ethyl acetate from 8:2 to 6:4. M.p. 111–113 °C. ^1^H-NMR (300 MHz, DMSO-*d*_6_) *δ* 1.01 (t, *J* = 7.1 Hz, 3H), 1.25 (t, *J* = 7.1 Hz, 3H), 3.74 (s, 3H), 3.93–4.03 (m, 2H), 4.20–4.30 (m, 2H), 4.34 (s, 1H), 6.89–6.94 (m, 2H), 7.07–7.12 (m, 2H), 7.17 (bs, 2H). ^13^C-APT-NMR (100 MHz, DMSO-*d*_6_) *δ* 13.5 (1C), 13.6 (1C), 38.2 (1C), 55.1 (1C), 56.7 (1C), 61.0 (1C), 62.3 (1C), 113.8 (1C), 114.0 (2C), 119.0 (1C), 128.7 (2C), 134.4 (1C), 134.4 (1C), 143.1 (1C), 158.4 (1C), 160.5 (1C), 164.0 (1C). IR (neat) (cm^−1^) ν 3,356, 2,984, 2,224, 1,733, 1,687, 1,642, 1,603, 1,570, 1513, 1,442, 1,369, 1,319, 1,277, 1,250, 1,179, 1,021, 833, 778, 512. HRMS (ESI+) calcd for C_19_H_20_N_2_O_6_Na 395.1214; found 395.1224 [M + Na].

#### Diethyl 6-amino-5-cyano-4-(*p*-tolyl)-4*H*-pyran-2,3-dicarboxylate (**3l**)

Following the general procedures, compound **3l** was obtained as a white solid in 21% yield (7.5 mg), 52% yield (18.5 mg) if 50 µL of EtOH are added, after 24 h of reaction at room temperature (route A); and in 50% yield (17.8 mg), after 2 h of reaction at room temperature (route B). The purification was performed by extraction with EtOAc (3 × 0.25 mL) and the column chromatography with *n-*hexane:ethyl acetate from 8:2 to 6:4. M.p. 128–130 °C. ^1^H-NMR (300 MHz, DMSO-*d*_6_) *δ* 1.00 (t, *J* = 7.1 Hz, 3H), 1.24 (t, *J* = 7.1 Hz, 3H), 2.27 (s, 3H), 3.92–4.02 (m, 2H), 4.20–4.30 (m, 2H), 4.34 (s, 1H), 7.03–7.06 (m, 2H), 7.14–7.18 (m, 4H). ^13^C-APT-NMR (75 MHz, DMSO-*d*_6_) *δ* 13.6 (1C), 13.7 (1C), 20.7 (1C), 38.6 (1C), 56.6 (1C), 61.2 (1C), 62.4 (1C), 113.5 (1C), 119.1 (1C), 127.5 (2C), 129.3 (2C), 136.7 (1C), 139.5 (1C), 143.5 (1C), 158.5 (1C), 160.6 (1C), 164.0 (1C). IR (neat) (cm^−1^) ν 3,411, 3,329, 3,272, 3,226, 3,199, 2,981, 2,924, 2,206, 2,109, 1,998, 1,738, 1,744, 1,709, 1,677, 1,649, 1,609, 1,370, 1,306, 1,288, 1,255, 1,171, 1,101, 1,040, 1,006, 749, 388. HRMS (ESI+) calcd for C_19_H_20_N_2_NaO_5_ 379.1264; found 379.1283 [M + Na].

#### Diethyl 6-amino-5-cyano-4-(pyridin-3-yl)-4*H*-pyran-2,3-dicarboxylate (**3m**)

Following the general procedures, compound **3m** was obtained as a white solid in 92% yield (31.6 mg), after 24 h of reaction at room temperature (route A); and in 88% yield (30.2 mg), after 2 h of reaction at room temperature (route B). The purification was performed by extraction with EtOAc (3 × 0.25 mL) and the column chromatography with *n-*hexane:ethyl acetate from 8:2 to 6:4. M.p. 142–144 °C. ^1^H-NMR (400 MHz, DMSO-*d*_6_) *δ* 0.98 (t, *J* = 7.1 Hz, 3H), 1.25 (t, *J* = 7.1 Hz, 3H), 3.98 (q, *J* = 7.1 Hz, 2H), 4.27 (q, *J* = 7.1 Hz, 2H), 4.51 (s, 1H), 7.30 (bs, 2H), 7.39–7.43 (m, 1H), 7.60–7.63 (m, 1H), 8.42 (d, *J* = 1.9 Hz, 1H), 8.49 (dd, *J* = 4.7, 1.6 Hz, 1H). ^13^C-APT-NMR (100 MHz, DMSO-*d*_6_) *δ* 13.5 (1C), 13.6 (1C), 36.5 (1C), 55.7 (1C), 61.2 (1C), 62.5 (1C), 112.3 (1C), 118.8 (1C), 124.0 (1C), 135.3 (1C), 138.1 (1C), 144.2 (1C), 148.7 (1C), 148.8 (1C), 158.6 (1C), 160.4 (1C), 163.7 (1C). IR (neat) (cm^−1^) ν 3,296, 2,983, 2,202, 2,160, 2,029, 1,977, 1,745, 1,719, 1,679, 1,617, 1,414, 1,368, 1,334, 1,292, 1,253, 1,182, 1,171, 1,097, 1,002, 857, 709, 611, 479. HRMS (ESI+) calcd for C_17_H_17_N_3_NaO_5_ 366.1060; found 366.1072 [M + Na].

#### Diethyl 6-amino-5-cyano-4-(4-(trifluoromethyl)phenyl)-4*H*-pyran-2,3-dicarboxylate (**3n**)

Following the general procedures, compound **3n** was obtained as a white solid in 34% yield (14.0 mg), after 24 h of reaction at room temperature (route A); and in 75% yield (30.8 mg), after 2 h of reaction at room temperature (route B). The purification was performed by extraction with EtOAc (3 × 0.25 mL) and the column chromatography with *n-*hexane:ethyl acetate from 8:2 to 6:4. M.p. 104–106 °C. ^1^H-NMR (300 MHz, DMSO-*d*_6_) *δ* 0.98 (t, *J* = 7.1 Hz, 3H), 1.25 (t, *J* = 7.1 Hz, 3H), 3.98 (q, *J* = 7.1 Hz, 2H), 4.27 (q, *J* = 7.1 Hz, 2H), 4.56 (s, 1H), 7.31 (bs, 2H), 7.43 (d, *J* = 7.9 Hz, 2H), 7.75 (d, *J* = 7.9 Hz, 2H). ^13^C-APT-NMR (75 MHz, DMSO-*d*_6_) *δ* 13.4 (1C), 13.6 (1C), 38.6 (1C), 55.8 (1C), 61.2 (1C), 62.5 (1C), 112.3 (1C), 118.8 (1C), 124.2 (q, *J* = 271.1 Hz, 1C), 125.6–125.7 (m, 2C), 128.1 (q, *J* = 31.6 Hz, 1C), 128.5 (2C), 144.3 (1C), 147.2 (1C), 158.5 (1C), 160.4 (1C), 163.7 (1C). IR (neat) (cm^−1^) ν 3,324, 3,095, 2,990, 2,234, 2,196, 1,953, 1,726, 1,676, 1,591, 1,565, 1,419, 1,319, 1,160, 1,115, 1,068, 1,014, 944, 849, 835, 621, 595, 386. HRMS (ESI+) calcd for C_19_H_17_F_3_N_2_NaO_5_ 433.0982; found 433.0964 [M + Na].

#### Diethyl 6-amino-4-(3,5-bis(trifluoromethyl)phenyl)-5-cyano-4*H*-pyran-2,3-dicarboxylate (**3o**)

Following the general procedures, compound **3o** was obtained as a white solid in 82% yield (39.2 mg), after 24 h of reaction at room temperature (route A); and in 79% yield (37.8 mg), after 2 h of reaction at room temperature (route B). The purification was performed by extraction with EtOAc (3 × 0.25 mL) and the column chromatography with *n-*hexane:ethyl acetate from 8:2 to 6:4. 176–178 °C. ^1^H-NMR (300 MHz, DMSO-*d*_6_) *δ* 0.93 (t, *J* = 7.1 Hz, 3H), 1.24 (t, *J* = 7.1 Hz, 3H), 3.96 (q, *J* = 7.1 Hz, 2H), 4.27 (q, *J* = 7.1 Hz, 2H), 4.86 (s, 1H), 7.41 (bs, 2H), 7.92 (bs, 2H), 8.09 (bs, 1H). ^13^C-APT-NMR (75 MHz, DMSO-*d*_6_) *δ* 13.3 (1C), 13.6 (1C), 38.3 (1C), 55.0 (1C), 61.3 (1C), 62.6 (1C), 112.1 (1C), 118.6 (1C), 121.5–121.7 (m, 1C), 123.2 (q, *J* = 272.8 Hz, 2C), 128.6–128.7 (m, 2C), 130.6 (q, *J* = 32.9 Hz, 2C), 144.0 (1C), 146.0 (1C), 158.7 (1C), 160.2 (1C), 163.7 (1C). IR (neat) (cm^−1^) ν 3,404, 3,323, 3,274, 3,202, 2,987, 2,199, 1,739, 1,714, 1,689, 1,650, 1,605, 1,372, 1,268, 1,165, 1,124, 1,091, 1,091, 1,045, 899, 779, 707, 680, 633, 492. HRMS (ESI+) calcd for C_20_H_16_F_6_N_2_NaO_5_ 501.0856; found 501.0871 [M + Na].

#### 5-Acetyl-2-amino-6-methyl-4-phenyl-4*H*-pyran-3-carbonitrile (**6**)

Following the general procedures, compound **6** was obtained as a white solid in 98% yield (24.9 mg), after 24 h of reaction at room temperature (route A); and in 95% yield (24.2 mg), after 2 h of reaction at room temperature (route B). The purification was performed by extraction with EtOAc (3 × 0.25 mL) and the column chromatography with *n-*hexane:ethyl acetate from 8:2 to 6:4. M.p. 226–228 °C. ^1^H-NMR (300 MHz, DMSO-*d*_6_) *δ* 2.06 (s, 3H), 2.24 (d, *J* = 1.0 Hz, 3H), 4.46 (d, *J* = 1.2 Hz, 1H), 6.85 (bs, 2H), 7.16–7.26 (m, 3H), 7.31–7.36 (m, 2H). ^13^C-APT-NMR (75 MHz, DMSO-*d*_6_) *δ* 18.5 (1C), 29.8 (1C), 38.8 (1C), 57.8 (1C), 115.0 (1C), 119.8 (1C), 127.0 (1C), 127.2 (2C), 128.8 (2C), 144.6 (1C), 154.8 (1C), 158.3 (1C), 198.4 (1C). IR (neat) (cm^−1^) ν 3,252, 3,116, 2,638, 2,242, 1,695, 1,666, 1,554, 1,456, 1,376, 1,357, 1,262, 1,173, 1,025, 745, 698, 546. HRMS (ESI+) calcd for C_15_H_14_N_2_O_2_Na 277.0947; found 277.0968 [M + Na].

#### Ethyl 6-amino-5-cyano-2-methyl-4-phenyl-4*H*-pyran-3-carboxylate (**7**)

Following the general procedures, compound **7** was obtained as a white solid in 97% yield (27.6 mg), after 24 h of reaction at room temperature (route A); and in 98% yield (27.9 mg), after 2 h of reaction at room temperature (route B). The purification was performed by extraction with EtOAc (3 × 0.25 mL) and the column chromatography with *n-*hexane:ethyl acetate from 8:2 to 6:4. M.p. 189–191 °C. ^1^H-NMR (300 MHz, DMSO-*d*_6_) *δ* 1.02 (t, *J* = 7.1 Hz, 3H), 2.31 (d, *J* = 1.0 Hz, 3H), 3.88–4.04 (m, 2H), 4.28 (s, 1H), 6.93 (bs, 2H), 7.12–7.15 (m, 2H), 7.18–7.24 (m, 1H), 7.29–7.33 (m, 2H). ^13^C-APT-NMR (100 MHz, DMSO-*d*_6_) *δ* 13.7 (1C), 18.1 (1C), 38.8 (1C), 57.3 (1C), 60.1 (1C), 107.2 (1C), 119.7 (1C), 126.8 (1C), 127.2 (2C), 128.4 (2C), 144.9 (1C), 156.6 (1C), 158.5 (1C), 165.4 (1C). IR (neat) (cm^−1^) ν 3,398, 3,326, 3,222, 3,201, 2,967, 2,188, 1,687, 1,674, 1,645, 1,608, 1,371, 1,255, 1,176, 1,058, 1,031, 831, 697, 475. HRMS (ESI+) calcd for C_16_H_16_N_2_O_3_Na 307.1053; found 307.1047 [M + Na].

#### Methyl 6-amino-5-cyano-2-methyl-4-phenyl-4*H*-pyran-3-carboxylate (**8**)

Following the general procedures, compound **8** was obtained as a white solid in 96% yield (25.9 mg), after 24 h of reaction at room temperature (route A); and in 97% yield (26.2 mg), after 2 h of reaction at room temperature (route B). The purification was performed by extraction with EtOAc (3 × 0.25 mL) and the column chromatography with *n-*hexane:ethyl acetate from 8:2 to 6:4. M.p. 166–168 °C. ^1^H-NMR (400 MHz, DMSO-*d*_6_) *δ* 2.31 (d, *J* = 0.8 Hz, 3H), 3.52 (s, 3H), 4.29 (bs, 1H), 6.90 (bs, 2H), 7.12–7.15 (m, 2H), 7.19–7.24 (m, 1H), 7.29–7.33 (m, 2H). ^13^C-APT-NMR (100 MHz, DMSO-*d*_6_) *δ* 18.2 (1C), 38.7 (1C), 51.5 (1C), 57.3 (1C), 107.2 (1C), 119.7 (1C), 126.8 (1C), 127.0 (2C), 128.5 (2C), 144.8 (1C), 156.8 (1C), 158.5 (1C), 166.0 (1C). IR (neat) (cm^−1^) ν 3,407, 3,328, 3,202, 2,953, 2,193, 1,697, 1,677, 1,645, 1,607, 1,406, 1,332, 1,261, 1,176, 1,120, 1,057, 951, 737, 695, 475. HRMS (ESI+) calcd for C_15_H_14_N_2_O_3_Na 293.0897; found 293.0883 [M + Na].

#### 2-Amino-7,7-dimethyl-5-oxo-4-phenyl-5,6,7,8-tetrahydro-4*H*-chromene-3-carbonitrile (**9**)

Following the general procedures, compound **9** was obtained as a white solid in 98% yield (28.9 mg), after 24 h of reaction at room temperature (route A); and in 98% yield (28.9 mg), after 2 h of reaction at room temperature (route B). The purification was performed by extraction with EtOAc (3 × 0.25 mL) and the column chromatography with *n-*hexane:ethyl acetate from 8:2 to 6:4. M.p. 218–220 °C. ^1^H-NMR (300 MHz, DMSO-*d*_6_) *δ* 0.95 (s, 3H), 1.04 (s, 3H), 2.10 (d, *J* = 16.1 Hz, 1H), 2.26 (d, *J* = 16.1 Hz, 1H), 2.46–2.58 (m, 2H), 4.17 (s, 1H), 7.02 (bs, 2H), 7.12–7.21 (m, 3H), 7.26–7.31 (m, 2H). ^13^C-APT-NMR (100 MHz, DMSO-*d*_6_) *δ* 26.8 (1C), 28.4 (1C), 31.8 (1C), 35.6 (1C), 39.7 (1C), 50.0 (1C), 58.3 (1C), 112.7 (1C), 119.6 (1C), 126.5 (1C), 127.1 (2C), 128.3 (2C), 144.7 (1C), 158.5 (1C), 162.4 (1C), 195.6 (1C). IR (neat) (cm^−1^) ν 3,383, 3,321, 3,209, 2,961, 2,198, 1,677, 1,657, 1,602, 1,369, 1,212, 1,248, 1,212, 1,138, 1,035, 694, 495. HRMS (ESI+) calcd for C_18_H_18_N_2_O_2_Na 317.1260; found 317.1272 [M + Na].

### Crystal structure determination

Crystals were mounted in inert oil on glass fibers and transferred to the cold gas stream of a Smart APEX CCD diffractometer equipped with a low-temperature attachment. Data were collected using monochromated MoKα radiation (λ = 0.71073 Å). Scan type ϖ. Absorption corrections based on multiple scans were applied using SADABS^126^. The structures were solved by direct methods and refined on F2 using the program SHELXT-2016^127^. All non-hydrogen atoms were refined anisotropically. CCDC deposition number 1982869 contains the supplementary crystallographic data. These data can be obtained free of charge by The Cambridge Crystallography Data Center.

### Biological assays

Calf thymus DNA was purchased from SigmaAldrich. DNA solutions were prepared to dissolve the solid ctDNA in a buffer solution of Tris (*tris*(hydroxymethyl)aminomethane)/HCl (0.1 M, pH 7.2) at room temperature to a final concentration of 1 mg/mL, leaving the mixture stirring overnight. The purity of the DNA was determined by measuring the absorbance ratio at A260 nm/A280 nm, being in all cases between 1.8 and 1.9, no further purification was needed. The molar concentration of the solution was determined by using a mean extinction coefficient of 6600 M^−1^ cm^−1^ for a single nucleotide at 260 nm.

### UV–Vis measurements

UV spectra were recorded using a Thermo Fisher Scientific Evolution 600 UV–Visible Spectrophotometer with 1 × 1 cm quartz cuvettes at 220–650 nm and 298 K. Stock solutions of the compounds **3a–o**, **6–9** were prepared in DMSO to a final concentration of 0.1 M. The titration experiments performed to obtain the binding constants were conducted as follows. First, an intermedium solution of the compound must be prepared in DMSO to a final concentration of 2 mM. Then, the assay solution of the selected compound must be prepared in 2 mL with the Tris/HCl buffer to a final concentration of 20 µM (20 µL of the intermedium solution and 1,980 µL of the buffer solution) and its UV–Vis spectra must be recorded to obtain its extinction coefficient (a baseline correction must be done using the corresponding dimethyl sulfoxide (DMSO) solution in the Tris/HCl buffer). Then, small portions of the ctDNA solution must be added to both assay and reference cuvettes to correct the final spectra with a mixture time of 10 min after every addition (4 × 5 µL, 4 × 10 µL, 1 × 20 µL).

### Viscosity measurements

Viscosity measurements were performed using a Cannon–Fenske viscometer (Afora, model 5354/2.50 series), submerged in a thermostatic bath at 298 K. The flow time was measured using a digital stopwatch. Each measure was repeated at least 4 times to obtain a mean time. The experiment was conducted as follows. 3.8 mL of a ctDNA solution in a buffer solution of Tris/HCl (0.1 M, pH 7.2) (1.14 mM measured by UV–Vis) was tested in the viscometer after 10 min to stabilize the temperature. Afterward, successive additions of 15 µL of a solution of **3n** 0.1 M were performed and the resulting mixture measured, taking the same precautions from before, with concentrations corresponding to 0.393 mM, 0.783 mM and 1.17 mM. Data is represented as ƞ/ƞ0 *vs* the ratio of **3n** to DNA concentration, being ƞ and ƞ0 the viscosity of the DNA solution with and without **3n**.

### Circular dichroism (CD)

Circular dichroism (CD) measurements were recorded on a Jasco J-810 spectropolarimeter with a 1 cm path length quartz cuvette, using a scanning speed of 200 nm/min and a spectral bandwidth of 10 nm, each spectrum is the mean of 4 scans. The experiments were conducted at 298 K, covering the 240–300 nm range. The titration procedure starts by measuring a solution of ctDNA 28 µM in a buffer solution of Tris/HCl (0.1 M, pH 7.2), with a baseline correction of the buffer. Then, two samples more were measured containing also 30 µM and 60 µM of **3n**, corrected with their respective baselines of **3n** and buffer.

### Fluorescence quenching competitive assays

Fluorescence experiments were recorded in Jobin–Yvon–Horiba Fluorolog FL3-11 spectrometer using a 1 cm path length quartz cuvette. Excitation wavelengths for ethidium bromide, methyl green and Hoechst 33342 are 525, 633 and 343 nm, respectively. Titration experiments were recorded at 298 K. ctDNA solution in Tris/HCl (0.1 M, pH 7.2) was prepared beforehand with a concentration of 1 mg/mL and its molar concentration was measured using a Thermo Fisher Scientific Evolution 600 UV–visible Spectrophotometer resulting in 1.13 mM.

Ethidium bromide and methyl green: ctDNA solutions were prepared in 2 mL Tris/HCl (0.1 M, pH 7.2) to a final concentration of 50 µM. The corresponding dye was added to a final concentration of 2.5 µM. Then, successive additions of 10 µL of a solution of compound **3n** [2 mM, Tris/HCl (0.1 M, pH 7.2)] were performed.

Hoechst 33342: ctDNA solution was prepared in 2 mL Tris/HCl (0.1 M, pH 7.2) to a final concentration of 200 µM. Hoechst 33342 was added to a final concentration of 2.5 µM. Then, successive additions of 1 µL of a solution of compound **3n** or **3m** [0.1 M, Tris/HCl (0.1 M, pH 7.2)] were performed.

## Supplementary information

Supplementary information
