# Supplementary material for: Ultrasound-assisted multicomponent synthesis of 4H-pyrans in water and DNA binding studies
Source: Sci Rep. 2020 Jul 14;10:11594. doi: 10.1038/s41598-020-68076-1 (PMC7360557; doi:10.1038/s41598-020-68076-1)
Supplement: Supplementary file 1 — Supplementary information [file 41598_2020_68076_MOESM1_ESM.docx]

**Ultrasound-assisted multicomponent synthesis of 4*H*-pyrans in water and DNA binding studies**

**Fernando Auria-Luna,^1^ Vanesa Fernández-Moreira,^2^ Eugenia Marqués-López,^1^ M. Concepción Gimeno^2^ and Raquel P. Herrera*^1^**

*^1^ Laboratorio de Organocatálisis Asimétrica, Departamento de Química Orgánica. Instituto de Síntesis Química y Catálisis Homogénea (ISQCH), CSIC-Universidad de Zaragoza. C/ Pedro Cerbuna 12, E-50009 Zaragoza, Spain.* [*raquelph@unizar.es*](mailto:raquelph@unizar.es)

*^2^ Departamento de Química Inorgánica. Instituto de Síntesis Química y Catálisis Homogénea (ISQCH), CSIC-Universidad de Zaragoza. C/ Pedro Cerbuna 12, E-50009 Zaragoza, Spain.*

**Supporting Information**

**Table of Contents**

**1.- Screening of the reaction conditions S2**

**2.- NMR spectra of compounds 3a-o, 6-9 S4**

**3.- UV-Vis spectra of compounds 3a-o, 6-9 S25**

**4.- Binding constant calculation *K*_b_ S35**

**5.- Viscosity measurements S36**

6.- Fluorescence experiments S37

**1.- Screening of the reaction conditions**

**Table S1. Screening of the reaction conditions for route A.**

| Entry | BnMN **2a**  (mmol) | DEOA **1a**  (mmol) | Cat.  (mol%) | Solvent  (mL) | Temp.  (ºC) | Time  (h) | Yield  (%) |
| --- | --- | --- | --- | --- | --- | --- | --- |
| 1 | 0.1 | 0.1 | NEt_3_ (20) | toluene 0.5 | r.t. | 24 | 63 |
| 2 | 0.1 | 0.1 | DIPEA (20) | toluene 0.5 | r.t. | 24 | 73 |
| 3 | 0.1 | 0.1 | DABCO (20) | toluene 0.5 | r.t. | 24 | 54 |
| 4 | 0.1 | 0.1 | piperidine (20) | toluene 0.5 | r.t. | 24 | 52 |
| 5 | 0.1 | 0.1 | pyridine (20) | toluene 0.5 | r.t. | 24 | 0 |
| 6 | 0.1 | 0.1 | - | toluene 0.5 | r.t. | 24 | 0 |
| 7 | 0.1 | 0.1 | NEt_3_ (20) | toluene 0.5 | r.t. | 10 min | 54 |
| 8 | 0.1 | 0.1 | NEt_3_ (20) | toluene 0.5 | r.t. | 1 | 89 |
| 9 | 0.1 | 0.1 | NEt_3_ (20) | Et_2_O 0.5 | r.t. | 1 | 67 |
| 10 | 0.1 | 0.1 | NEt_3_ (20) | CH_2_Cl_2_ 0.5 | r.t. | 1 | 67 |
| 11 | 0.1 | 0.1 | NEt_3_ (20) | CHCl_3_ 0.5 | r.t. | 1 | 71 |
| 12 | 0.1 | 0.1 | NEt_3_ (20) | hexane 0.5 | r.t. | 1 | 0 |
| 13 | 0.1 | 0.1 | NEt_3_ (20) | THF 0.5 | r.t. | 1 | 69 |
| 14 | 0.1 | 0.1 | NEt_3_ (20) | acetone 0.5 | r.t. | 1 | 70 |
| 15 | 0.1 | 0.1 | NEt_3_ (20) | DMF 0.5 | r.t. | 1 | 75 |
| 16 | 0.1 | 0.1 | NEt_3_ (20) | xylene 0.5 | r.t. | 1 | 77 |
| 17 | 0.1 | 0.1 | NEt_3_ (20) | EtOH 0,5 | r.t. | 1 | 84 |
| 18 | 0.1 | 0.1 | NEt_3_ (20) | AcOEt 0.5 | r.t. | 1 | 67 |
| 19 | 0.1 | 0.1 | NEt_3_ (20) | MeCN 0.5 | r.t. | 1 | 93 |
| 20 | 0.1 | 0.1 | NEt_3_ (20) | toluene 0.5 | r.t. | 1 | 90 |
| 21 | 0.1 | 0.1 | NEt_3_ (20) | MeOH 0.5 | r.t. | 1 | 95 |
| 22 | 0.1 | 0.1 | DIPEA (20) | MeOH 0.5 | r.t. | 1 | 47 |
| 23 | 0.1 | 0.1 | _ | _ | r.t. | 1 | 0 |
| 24 | 0.1 | 0.1 | NEt_3_ (20) | _ | r.t. | 1 | 47 |
| 25 | 0.1 | 0.1 | DIPEA (20) | _ | r.t. | 1 | 54 |
| 26 | 0.1 | 0.1 | NEt_3_ (10) | MeOH 0.5 | r.t. | 1 | 64 |
| 27 | 0.1 | 0.1 | DIPEA (10) | MeOH 0.5 | r.t. | 1 | 48 |
| 28 | 0.1 | 0.1 | NEt_3_ (5) | MeOH 0.5 | r.t. | 1 | 49 |
| 29 | 0.1 | 0.1 | DIPEA (5) | MeOH 0.5 | r.t. | 1 | 26 |
| 30 | 0.1 | 0.1 | - | H_2_O 0.5 | r.t. | 48 | 26 |
| 31 | 0.1 | 0.1 | NEt_3_ (20) | H_2_O 0.5 | r.t. | 48 | 43 |
| 32 | 0.1 | 0.1 | NEt_3_ (20) | H_2_O 0.25 | r.t. | 24 | 59 |
| 33 | 0.1 | 0.1 | NEt_3_ (20) | H_2_O 0.25 | r.t. | 12 | 55 |
| 34 | 0.1 | 0.1 | NEt_3_ (20) | H_2_O 0.25 | r.t. | 6 | 51 |
| 35 | 0.1 | 0.1 | NEt_3_ (20) | H_2_O 0.25 | r.t. | 3 | 45 |
| 36 | 0.1 | 0.1 | NEt_3_ (20) | H_2_O 0.25 | r.t. | 1 | 18 |
| 37 | 0.1 | 0.1 | NEt_3_ (20) | H_2_O 0.25 | r.t. | 0.5 | 0 |
| 38 | 0.1 | 0.2 | NEt_3_ (20) | H_2_O 0.25 | r.t. | 24 | 85 |

**Table S2. Screening of the reaction conditions for route B.**

| Entry | DEOA **1a** (mmol) | Aldeh. **4a** (mmol) | MN **5a** (mmol) | Ultrasound | Time (h) | Yield (%) |
| --- | --- | --- | --- | --- | --- | --- |
| 1 | 0.1 | 0.1 | 0.1 | 40 kHz | 2 | 92 |
| 2 | 0.1 | 0.1 | 0.1 | _ | 2 | 10 |

**2.- NMR Spectra of Compounds 3a-o, 6-9**

**Figure S1. ^1^H and ^13^C-APT NMR spectra of diethyl 6-amino-5-cyano-4-phenyl-4*H*-pyran-2,3-dicarboxylate (3a)**

**Figure S2. ^1^H and ^13^C-APT NMR spectra of diethyl 6-amino-4-(4-chlorophenyl)-5-cyano-4*H*-pyran-2,3-dicarboxylate (3b)**

**Figure S3. ^1^H and ^13^C-APT NMR spectra of diethyl 6-amino-4-(3-chlorophenyl)-5-cyano-4*H*-pyran-2,3-dicarboxylate (3c)**

**Figure S4. ^1^H and ^13^C-APT NMR spectra of diethyl 6-amino-4-(4-bromophenyl)-5-cyano-4*H*-pyran-2,3-dicarboxylate (3d)**

**Figure S5. ^1^H and ^13^C-APT NMR spectra of diethyl 6-amino-5-cyano-4-(4-nitrophenyl)-4*H*-pyran-2,3-dicarboxylate (3e)**

**Figure S6. ^1^H and ^13^C-APT NMR spectra of diethyl 6-amino-5-cyano-4-(3-nitrophenyl)-4*H*-pyran-2,3-dicarboxylate (3f)**

**Figure S7. ^1^H and ^13^C-APT NMR spectra of diethyl 6-amino-5-cyano-4-(4-cyanophenyl)-4*H*-pyran-2,3-dicarboxylate (3g)**

**Figure S8. ^1^H, ^13^C-APT, ^13^C-HSQC, ^13^C-HMBC NMR spectra of diethyl 6-amino-5-cyano-4-(naphthalen-1-yl)-4*H*-pyran-2,3-dicarboxylate (3h)**

**Figure S9. ^1^H and ^13^C-APT NMR spectra of diethyl 6-amino-5-cyano-4-(furan-2-yl)-4*H*-pyran-2,3-dicarboxylate (3i)**

**Figure S10. ^1^H and ^13^C-APT NMR spectra of diethyl 6-amino-5-cyano-4-(thiophen-2-yl)-4*H*-pyran-2,3-dicarboxylate (3j)**

**Figure S11. ^1^H and ^13^C-APT NMR spectra of diethyl 6-amino-5-cyano-4-(4-methoxyphenyl)-4*H*-pyran-2,3-dicarboxylate (3k)**

**Figure S12. ^1^H and ^13^C-APT NMR spectra of diethyl 6-amino-5-cyano-4-(*p*-tolyl)-4*H*-pyran-2,3-dicarboxylate (3l)**

**Figure S13. ^1^H and ^13^C-APT NMR spectra of diethyl 6-amino-5-cyano-4-(pyridin-3-yl)-4*H*-pyran-2,3-dicarboxylate (3m)**

**Figure S14. ^1^H and ^13^C-APT NMR spectra of diethyl 6-amino-5-cyano-4-(4-(trifluoromethyl)phenyl)-4H-pyran-2,3-dicarboxylate (3n)**

**Figure S15. ^1^H and ^13^C-APT NMR spectra of diethyl 6-amino-4-(3,5-bis(trifluoromethyl)phenyl)-5-cyano-4H-pyran-2,3-dicarboxylate (3o)**

**Figure S16. ^1^H and ^13^C-APT NMR spectra of 5-acetyl-2-amino-6-methyl-4-phenyl-4*H*-pyran-3-carbonitrile (6)**

**Figure S17. ^1^H and ^13^C-APT NMR spectra of ethyl 6-amino-5-cyano-2-methyl-4-phenyl-4*H*-pyran-3-carboxylate (7)**

**Figure S18. ^1^H and ^13^C-APT NMR spectra of methyl 6-amino-5-cyano-2-methyl-4-phenyl-4*H*-pyran-3-carboxylate (8)**

**Figure S19. ^1^H and ^13^C-APT NMR spectra of 2-amino-7,7-dimethyl-5-oxo-4-phenyl-5,6,7,8-tetrahydro-4*H*-chromene-3-carbonitrile (9)**

**3.- UV-Vis spectra of compounds 3a-o, 6-9**

**Figure S20.** Titration experiment of diethyl 6-amino-5-cyano-4-phenyl-4*H*-pyran-2,3-dicarboxylate (**3a**)

**

**

**Figure S21.** Titration experiment of diethyl 6-amino-4-(4-chlorophenyl)-5-cyano-4*H*-pyran-2,3-dicarboxylate (**3b**)

**

**

**Figure S22.** Titration experiment of diethyl 6-amino-4-(3-chlorophenyl)-5-cyano-4*H*-pyran-2,3-dicarboxylate (**3c**)

**

**

**Figure S23.** Titration experiment of diethyl 6-amino-4-(4-bromophenyl)-5-cyano-4*H*-pyran-2,3-dicarboxylate (**3d**)

**

**

**Figure S24.** Titration experiment of diethyl 6-amino-5-cyano-4-(4-nitrophenyl)-4*H*-pyran-2,3-dicarboxylate (**3e**)

**

**

**Figure S25.** Titration experiment of diethyl 6-amino-5-cyano-4-(3-nitrophenyl)-4*H*-pyran-2,3-dicarboxylate (**3f**)

**

**

**Figure S26.** Titration experiment of diethyl 6-amino-5-cyano-4-(4-cyanophenyl)-4*H*-pyran-2,3-dicarboxylate (**3g**)

**

**

**Figure S27.** Titration experiment of diethyl 6-amino-5-cyano-4-(naphthalen-1-yl)-4*H*-pyran-2,3-dicarboxylate (**3h**)

**

**

**Figure S28.** Titration experiment of diethyl 6-amino-5-cyano-4-(furan-2-yl)-4*H*-pyran-2,3-dicarboxylate (**3i**)

**

**

**Figure S29.** Titration experiment of diethyl 6-amino-5-cyano-4-(thiophen-2-yl)-4*H*-pyran-2,3-dicarboxylate (**3j**)

**

**

**Figure S30.** Titration experiment of diethyl 6-amino-5-cyano-4-(4-methoxyphenyl)-4H-pyran-2,3-dicarboxylate (**3k**)

**

**

**Figure S31.** Titration experiment of diethyl 6-amino-5-cyano-4-(p-tolyl)-4*H*-pyran-2,3-dicarboxylate (**3l**)

**

**

**Figure S32.** Titration experiment of diethyl 6-amino-5-cyano-4-(pyridin-3-yl)-4*H*-pyran-2,3-dicarboxylate (**3m**)

**

**

**Figure S33.** Titration experiment of diethyl 6-amino-5-cyano-4-(4-(trifluoromethyl)phenyl)-4H-pyran-2,3-dicarboxylate (**3n**)

**

**

**Figure S34.** Titration experiment of diethyl 6-amino-4-(3,5-bis(trifluoromethyl)phenyl)-5-cyano-4H-pyran-2,3-dicarboxylate (**3o**)

**

**

**Figure S35.** Titration experiment of 5-acetyl-2-amino-6-methyl-4-phenyl-4*H*-pyran-3-carbonitrile (**6**)

**

**

**Figure S36.** Titration experiment of ethyl 6-amino-5-cyano-2-methyl-4-phenyl-4*H*-pyran-3-carboxylate (**7**)

**

**

**Figure S37.** Titration experiment of methyl 6-amino-5-cyano-2-methyl-4-phenyl-4*H*-pyran-3-carboxylate (**8**)




**Figure S38.** ^1^H and ^13^C-APT NMR spectra of 2-amino-7,7-dimethyl-5-oxo-4-phenyl-5,6,7,8-tetrahydro-4*H*-chromene-3-carbonitrile (**9**)




**4.- Binding constant calculation *K*_b_**

A ctDNA solution is prepared at 1 mg/mL in a tris (*tris*(hydroxymethyl)aminomethane)/HCl (0.1 M, pH 7.2) buffer. Then, the purity of the ctDNA is checked recording its spectra and calculating the absorption ratio between 260 and 280 nm, being optimal in a range of 1.8-1.9 to ensure that DNA is sufficiently free of protein.^[[1]](#footnote-1)^ This experiment also provides the means to calculate the concentration (M) of ctDNA using the Beer-Lambert formula with an extinction coefficient of 6600 M^-1^cm^-1^ for a single nucleotide at 260 nm and 1 cm path length.^[[2]](#footnote-2)^ Every experiment is corrected with a baseline of the buffer and the corresponding concentration of dimethyl sulfoxide (DMSO). The additions of the ctDNA solution are performed in both assay and reference cuvettes, to only observe the spectra of the corresponding pyran.

Then, the binding constant was calculated for all 4*H*-pyrans using the modified Benesi-Hildebrand equation:^[[3]](#footnote-3),^^[[4]](#footnote-4)^

$$\frac{\left| DNA \right|}{\varepsilon_{f}- \varepsilon_{a}}= \frac{\left| DNA \right|}{\varepsilon_{f}- \varepsilon_{b}}+ \frac{1}{K_{b}\left( \varepsilon_{f}- \varepsilon_{b} \right)}$$

***K_b_*** is the binding constant, **ε_f,_ ε_a_** and **ε_b_** are the extinction coefficients for the free compound, the apparent coefficient (calculated as the ratio of the absorbance and the concentration of the compound in each experiment) and the compound-DNA complex, respectively. Plotting **[DNA] /(ε_f_ - ε_a_)** *vs* **[DNA]** for every experiment and performing a lineal fitting gives as a result a line equation **y = mx + n** in which **m/n** is the binding constant, as shown in Fig. S41.

**
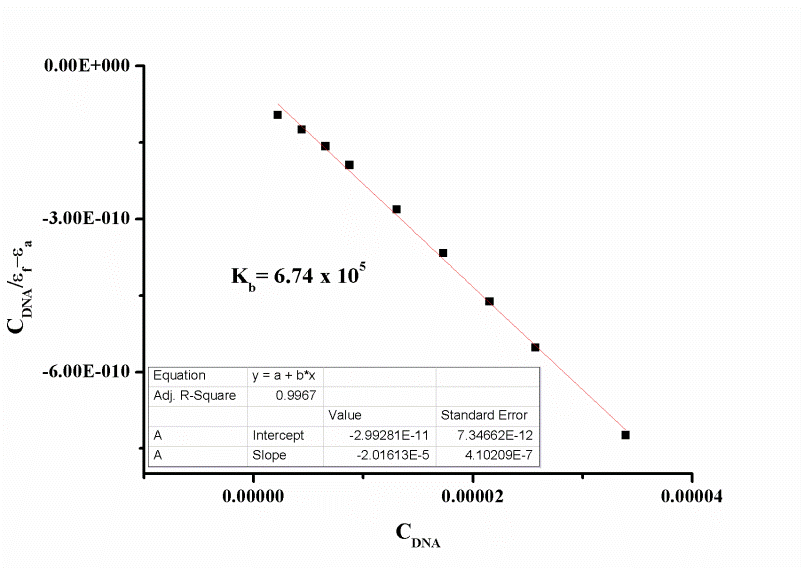
**

**Figure S39.** Binding constant (*K*_b_) calculation for **3n** using data from Figure S33.

**5.- Viscosity measurements**

**Figure S40.** Four viscosity measurements increasing the concentration of compound **3n** in a ratio **3n/**DNA from 0 to 1/3, 2/3 and 1 and maintaining the DNA concentration constant (1.14 mM). Viscosity is calculated as the ratio of the measurement in each experiment regarding the initial value. Each value is the mean ± SD of 3 experiments.

6.- Fluorescence experiments





Figure S41. Excitation and emission profiles of Hoechst 33342 (H33342), methyl green (MeGr), ethidium bromide (EtBr) and compound 3n. All spectra recorded with a solution of ctDNA 50 µM and the compound at 5 µM. All spectra have been normalized to 1.

1. Wilfinger, W. W., Mackey, K. & P. Chomczynski, P. Effect of pH and Ionic Strength on the Spectrophotometric Assessment of Nucleic Acid Purity. *Biotechniques* **22**, 474–481, DOI: <https://doi.org/10.2144/97223st01> (1997). [↑](#footnote-ref-1)
2. Kumar, C. V. & Asuncion, E. H. DNA binding studies and site selective fluorescence sensitization of an anthryl probe. *J. Am. Chem. Soc*. **115**, 8547–8553, DOI: <https://doi.org/10.1021/ja00072a004> (1993). [↑](#footnote-ref-2)
3. Benesi, H. A. & Hildebrand, J. H. A Spectrophotometric Investigation of the Interaction of Iodine with Aromatic Hydrocarbons. *J. Am. Chem. Soc.* **71**, 2703–2707, DOI: <https://doi.org/10.1021/ja01176a030> (1949). [↑](#footnote-ref-3)
4. (a) Wolfe, A., Shimer, G. H. & Meehan, T. Polycyclic Aromatic Hydrocarbons Physically Intercalate Into Duplex Regions of Denatured DNA. *Biochemistry* **26**, 6392–6396, DOI: <https://doi.org/10.1021/bi00394a013> (1987); (b) Gamov, G. A., Zavalishin, M. N., Sharnin, V. A. Comment on the frequently used method of the metal complex-DNA binding constant determination from UV–Vis data. *Spectrochim. Acta A Mol. Biomol. Spectrosc.* **206**, 160–164, DOI: <https://doi.org/10.1016/j.saa.2018.08.009> (2019). [↑](#footnote-ref-4)
